# *Lactobacillus rhamnosus* GG induces STING-dependent IL-10 in intestinal monocytes and alleviates inflammatory colitis in mice

**DOI:** 10.1172/JCI174910

**Published:** 2025-02-03

**Authors:** Wei Si, Xin Zhao, Ruitong Li, Yaopeng Li, Cui Ma, Xiaohan Zhao, Jason Bugno, Yuchang Qin, Junmin Zhang, Hongwei Liu, Liangliang Wang

**Affiliations:** 1 State Key Laboratory of Animal Nutrition, Institute of Animal Sciences of Chinese Academy of Agricultural Sciences, Beijing, China.; 2 Department of Animal Science, McGill University, Montreal, Quebec, Canada.; 3 Pritzker School of Molecular Engineering and; 4 Department of Radiation and Cellular Oncology, University of Chicago, Chicago, Illinois, USA.; 5 The Laboratory of Microbiome and Microecological Technology, Institute of Microbiology, Chinese Academy of Sciences, Beijing, China.

**Keywords:** Immunology, Inflammation, Inflammatory bowel disease, Innate immunity, Monocytes

## Abstract

Preclinical and clinical observations indicate that the probiotic *Lactobacillus rhamnosus* GG (LGG) can modulate colonic inflammation. However, the underlying mechanisms have not been explored in depth. Here, we demonstrate that oral administration of live LGG alleviated inflammatory colitis by increasing IL-10 expression in intestinal Ly6C^+^ monocytes. Mechanistically, LGG induced IL-10 production via the stimulator of IFN genes (STING)/TBK1/NF-κB (RELA) signaling pathway in intestinal Ly6C^+^ monocytes, enhancing their immune-suppressive function. Elevated IL-10 subsequently activated IL-10 signaling in Ly6C^+^ monocytes, resulting in an IL-10–based autocrine regulatory loop and inhibition of proinflammatory cytokine production. Furthermore, LGG shifted the gut microbial community and its metabolic functions, leading to intestinal immune responses against colitis. Fecal microbiota transplantation from LGG-colonized mice alleviated immune checkpoint blockade–associated colitis. Our findings highlight the importance of STING signaling in IL-10–dependent antiinflammatory immunity and establish an empirical basis for developing oral administration of live LGG as an efficient and safe therapeutic strategy against inflammatory colitis.

## Abstract 2

Lactobacillus rhamnosus GG alleviates inflammatory colitis requiring STING/RELA axis-induced IL-10 production in intestinal Ly6C+ monocytes and the shifts in gut microbiota and their metabolic function.

## Graphical abstract

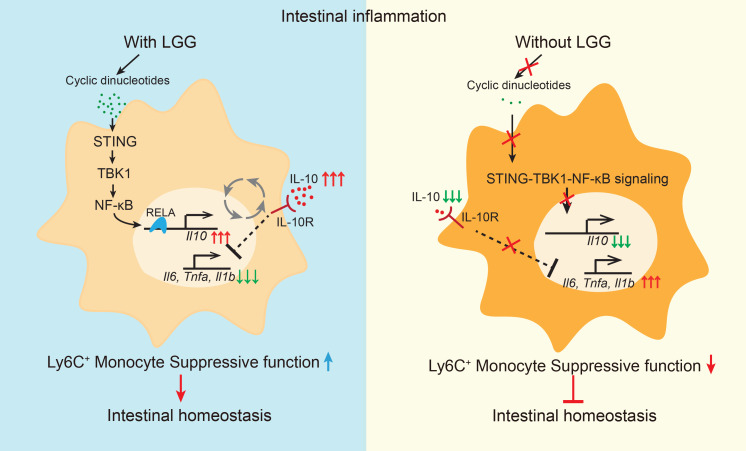

## Introduction

Human inflammatory bowel disease (IBD) encompasses two conditions (Crohn’s disease and ulcerative colitis) that are characterized by chronic inflammation of the gastrointestinal tract (1, 2). Current treatment regimens for IBD largely work through cytokine modulation or inflammatory immune cell targeting (3). Despite continuous technological and therapeutic improvements, most IBD therapeutics are associated with serious side effects. There is an urgent need for better and safer agents/therapies to improve outcomes for patients with IBD. Recent studies have identified a correlation between the gut microbiome, probiotics, and human IBD (4). However, most therapeutic approaches are in early development, highlighting the need for next-generation probiotics as therapeutic agents that can alter the gut microbiota and relieve gut inflammation.

*Lactobacillus rhamnosus* GG (LGG) is one of the most widely used and studied probiotics owing to its favorable safety profile and efficacy. Several studies have demonstrated the remarkable effects of LGG in preventing ulcerative colitis in both the clinical setting (5) and murine models of colitis (6, 7). Although most of these studies focused on the effects of LGG on epithelial cells rather than immune cells in the gut (8), it has become more apparent that LGG controls intestinal homeostasis through suppressing inflammation (9). In seeming contrast, we and others previously demonstrated that oral administration of live LGG can activate DCs to orchestrate a proinflammatory response in the condition of infection or cancer (10, 11). Given these conflicting reports, it is critical to investigate the role of the gut immune response in LGG-alleviated colitis and clarify the specific molecular mechanisms.

Mounting evidence highlights the importance of IL-10 and its signaling in the inhibition of proinflammatory cytokines in both human and mouse colitis (12). Mice deficient in IL-10 have increased proinflammatory cytokine production and develop severe colitis (13, 14). In a particular study, mononuclear phagocytes (MNPs), a population of myeloid cells, including monocytes, macrophages, and DCs, has been reported to produce IL-10 in the context of colitis (15). It has been suggested that LGG alleviation of colitis likely relies on elevated IL-10 in the intestine (16) but with unknown cellular source and the underlying mechanisms. Motivated by these emerging insights, we hypothesized that LGG might induce IL-10 in specific intestinal myeloid cells to prevent colitis.

The stimulator of IFN genes (STING) and its signaling play a critical role in regulating intestinal immunity. STING in MNPs predominantly controls IL-10 expression in the prevention of inflammatory colitis (15), and treatment with STING agonists inhibits intestinal inflammation in mice (17). On the contrary, STING activation has also been linked to colitis exacerbation (18, 19). We reasoned that STING activation of a proinflammatory or antiinflammatory response may be context dependent during colitis, yet little is known regarding the molecular basis of the antiinflammatory activities of STING.

In this study, we sought to evaluate the protective activity of LGG in inflammatory colitis and identify the molecular mechanisms. Oral administration of live LGG effectively alleviated dextran sodium sulfate–induced (DSS-induced) intestinal inflammation colitis through IL-10 upregulation in intestinal Ly6C^+^ monocytes. Mechanistically, LGG induced IL-10 expression via the STING/TBK1/RELA signaling pathway. We also report the existence of an IL-10–based autocrine regulatory loop induced by LGG in the context of colitis. Metabolic profiling and 16s rRNA sequencing revealed an enrichment of 3 gut bacterial genera after LGG treatment that contributed to the alleviation of intestinal inflammation, confirmed by the fecal microbiota transplantation (FMT). Our findings provide important insights into the mechanisms by which LGG interacts with STING/IL-10 in monocytes to control antiinflammatory immunity against inflammatory colitis.

## Results

### Oral administration of live LGG alleviates inflammatory colitis in an IL-10–dependent manner.

**Figure 1:**
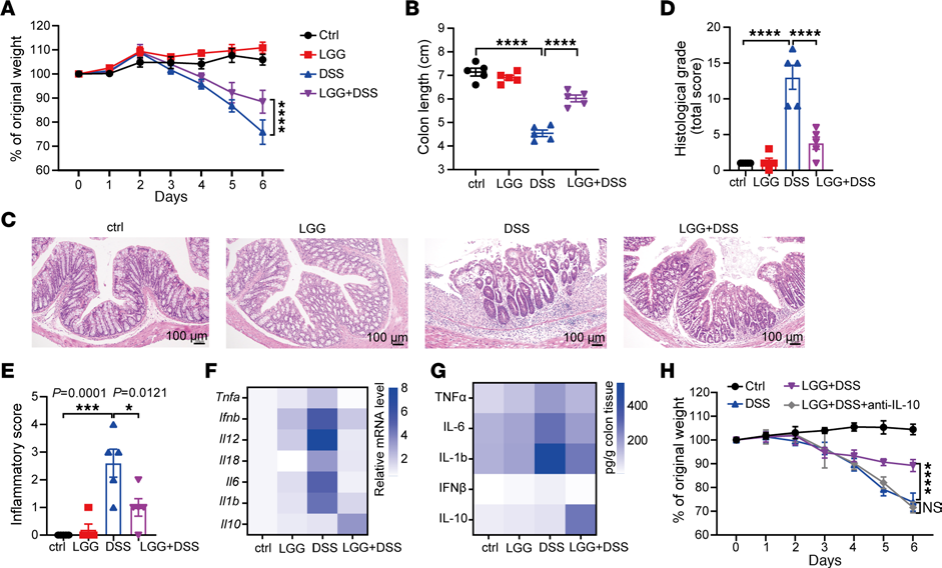
Oral administration of live LGG alleviates inflammatory colitis in a manner dependent on IL-10. (**A**) Body weight assessment of WT SPF mice receiving 3% of dextran sodium sulfate (DSS) in drinking water for 7 days. WT mice were orally administered LGG (2 × 10^9^ CFU) daily for 2 weeks (starting 1 week before DSS administration). The body weight of mice was measured every day. The percentage weight change is shown (*n* = 5 per group). (**B**) Colon length on day 7 of each group, as indicated in **A** (*n* = 5 per group). (**C**–**E**) Representative images of H&E-stained colon tissues (**C**) and the matching histological grade (**D**) and inflammation score (**E**) in mice with different treatments as indicated (day 7) (*n* = 5 per group). Scale bar: 100 μm. (**F**) Heatmap showing the mRNA expression of proinflammatory cytokines and antiinflammatory cytokine genes (identified by qPCR analysis) in colon tissues of each group, as indicated in **A** (*n* = 3 per group). (**G**) Heatmap showing the protein levels of the matching proinflammatory cytokines and antiinflammatory cytokines (identified by qPCR analysis) as in **F** (*n* = 3 per group). (**H**) Body weight assessment of WT SPF mice receiving the indicated treatment (*n* = 5). One hundred micrograms of anti–IL-10 antibody was administered i.p. every other day during DSS treatment (*n* = 5 per group). Data are expressed as mean ± SEM. One of 2 or 3 representative experiments is shown. Statistical analysis was performed using 2-way ANOVA test with corrections for multiple variables (**A** and **H**) and 1-way ANOVA with Bonferroni’s multiple comparison tests (**B**, **D**, and **E**). *P < 0.05, ***P < 0.001, ****P < 0.0001.

To evaluate the therapeutic effects of LGG against inflammatory colitis, we employed a DSS-induced acute colitis model (20) and orally treated specific pathogen–free (SPF) mice with live LGG starting 1 week before DSS administration. We observed a significant decrease in body weight and colon length in DSS-treated mice compared with nontreated mice (Figure 1, A and B), consistent with DSS-induced intestinal inflammation. Oral administration of LGG significantly improved weight loss and colon shortening induced by DSS (Figure 1, A and B). Of note, these effects were even more pronounced in germ-free (GF) mice (Supplemental Figure 1, A and B; supplemental material available online with this article; https://doi.org/10.1172/JCI174910DS1). We performed H&E staining on colon sections to assess the effects of LGG on histological and inflammatory injury. Mice administered with LGG in combination with DSS had significantly decreased histological grading and inflammatory scores compared with those given DSS alone (Figure 1, C–E). Furthermore, we evaluated the therapeutic efficacy of LGG treatment against a murine chronic 3-cycle DSS-induced colitis and a T cell transfer colitis induced by transferring naive CD4^+^ T cells into *Rag1*-KO mice and found that LGG treatment significantly improved weight loss in both two colitis models (Supplemental Figure 1, C and D). These findings demonstrate the therapeutic potential for LGG in alleviating colitis.

Live LGG had no significant effects on body weight, colon length, and histological and inflammatory injury when given alone (Figure 1, A–E), consistent with the favorable safety profile of LGG observed in both preclinical and clinical studies. Since others have demonstrated that inactivated probiotics, including LGG, can modulate immune responses in mice (21), we gavaged the same dose of heat-killed LGG in DSS-treated GF mice. We found that heat-killed LGG failed to improve either the body weight loss or colon length shortening (Supplemental Figure 1, A and B), consistent with our previous observation that inactivated LGG had no immune effects (11). Taken together, live LGG is required for its protective effects on colitis.

To probe the underlying immunological mechanisms behind LGG protective activity, we subsequently profiled the expression of inflammatory cytokines in colon tissues. The proinflammatory cytokines TNF-α, IL-6, IL-1b, and IFN-β were upregulated upon DSS treatment but were downregulated with LGG treatment at both the transcriptional and protein levels (Figure 1, F and G). Interestingly, LGG induced expression of the antiinflammatory cytokine IL-10 in DSS-treated mice (Figure 1, A and B). Similar changes in both pro- and antiinflammatory cytokines were observed in GF mice (Supplemental Figure 1, E and F). Given these results, we suspected that IL-10 was required for the protective activity of live LGG. To confirm this, we blocked IL-10 in LGG+DSS-treated mice using an IL-10–neutralizing antibody and found that the protective effect of LGG was abrogated in the absence of IL-10 (Figure 1H). These findings indicate that live LGG alleviates inflammatory colitis in an IL-10–dependent manner.

### Monocytic IL-10 is predominantly responsible for the LGG-mediated protective effect against colitis.

**Figure 2:**
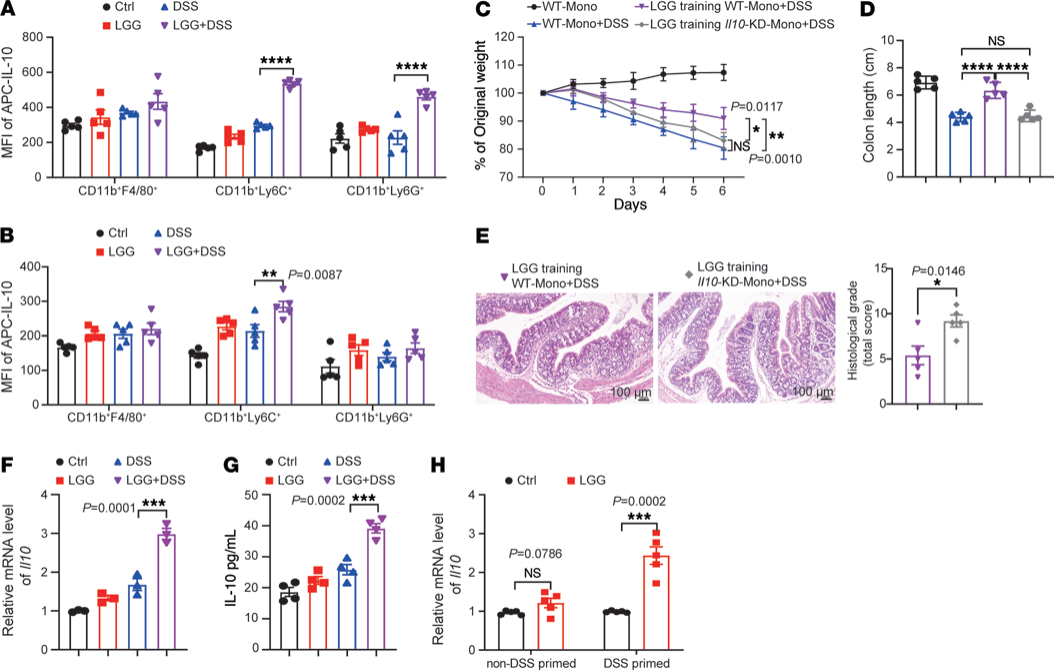
Monocytic IL-10 is predominantly responsible for the LGG protective activity against colitis. (**A** and **B**) Mean fluorescence intensity (MFI) of IL-10 in different myeloid cells (CD45^+^CD11b^+^F4/80^+^, CD45^+^CD11b^+^Ly6C^+^, and CD45^+^CD11b^+^Ly6G^+^) from MLNs of GF mice (**A**) or SPF mice (**B**) with the indicated treatment (*n* = 5 per group). (**C** and **D**) WT or *Il10*-knockdown (*Il10*-KD) bone marrow–derived monocytes with or without LGG treatment were used for adoptive transfer into 3% DSS–treated mice. Body weight was monitored (**C**). Colon length on day 6 of each group, as indicated in **C**. (**D**) (*n* = 5 per group). (**E**) Representative images of H&E-stained colon tissues and the matching histological grade score (*n* = 5 per group) in DSS-treated mice with adoptive transfer with LGG-training WT or *Il10*-KD monocytes. Scale bar: 50 μm. (**F**) Ly6C^+^ monocytes were isolated from MLNs in mice following the indicated treatment and subjected to qPCR analysis of the mRNA level of *Il10* (n = 3 per group). (**G**) IL-10 levels produced by Ly6C^+^ monocytes, as shown in **F** (*n* = 4 per group). (**H**) 2 × 10^5^ Ly6C^+^ monocytes were isolated from untreated or DSS-treated mice and cocultured with live LGG (2 × 10^6^ CFU) in vitro. The *Il10* mRNA levels were measured by qPCR (*n* = 5 per group). Data are expressed as mean ± SEM. One of 2 or 3 representative experiments is shown. Statistical analysis was performed using 1-way ANOVA with Bonferroni’s multiple comparison tests (**A**, **B**, **D**, **F**, and **G**), 2-way ANOVA test with corrections for multiple variables (**C**), and unpaired 2-tailed Student’s *t* tests (**E** and **H**). *P < 0.05, **P < 0.01, ***P < 0.001, ****P < 0.0001

Monocytes, macrophages, and helper T cells can express IL-10 (22). We performed flow cytometry analysis of mesenteric lymph nodes (MLNs) from GF mice treated with DSS and/or LGG to dissect the main source of IL-10. IL-10 was significantly upregulated in CD11b^+^Ly6C^+^ and CD11b^+^Ly6G^+^ cells after LGG+DSS treatment compared with cells after treatment with DSS alone, but this effect as not seen in macrophages or T helper cells (Figure 2A). Importantly, LGG had no effect on the expression of IL-10 in these immune cell populations isolated from either peripheral lymph nodes or spleens from DSS-treated or untreated mice (Supplemental Figure 2, A and B). In DSS-treated SPF mice, LGG treatment selectively induced more than a 2-fold increase in IL-10 expression in CD11b^+^Ly6C^+^ cells but not in other myeloid subsets, including the CD11b^+^Ly6G^+^ population (Figure 2B). In addition, we detected the level of IL-10 in different immune cells isolated from lamina propria and observed that IL-10 was significantly upregulated in Ly6C^+^ monocytes (CD11b^+^Ly6C^+^) after LGG+DSS treatment compared with DSS alone (Supplemental Figure 2C). These data suggest that Ly6C^+^ monocytes are the major source of IL-10 in LGG-mediated protection against DSS-induced colitis.

To further confirm the role of monocytic IL-10 in LGG-mediated protective effects during colitis, we knocked down *Il10* using siRNA in bone marrow–derived monocytes to generate *Il10*-KD BM monocytes (Supplemental Figure 2D). WT and *Il10*-KD BM monocytes were then treated with live LGG in vitro before adoptive transfer and DSS treatment. Either WT or *Il10*-KD monocytes indeed migrated to the MLN upon different treatments (Supplemental Figure 2E), and the *Il10* mRNA level was still significantly decreased in isolated LGG-training *Il10*-KD monocytes compared with LGG-training WT monocytes upon DSS treatment (Supplemental Figure 2F). Adoptive transfer of LGG-training WT bone marrow monocytes into DSS-treated mice resulted in a significant improvement in both body weight and colon length compared with those of their nontrained counterparts (Figure 2, C and D). These protective effects were completely ablated in mice adoptively transferred LGG-training *Il10*-KD BM monocytes (Figure 2, C and D). Histological examination of colon tissue from DSS-treated mice further demonstrated that mice adoptively transferred *Il10*-KD BM monocytes had a diminished recovery from intestinal inflammation compared with mice adoptively transferred LGG-training WT bone marrow monocytes (Figure 2E). Taken together, these findings highlight the critical role of monocytic IL-10 in LGG-mediated colitis control.

Of interest was that the population of monocytes in MLNs did not significantly change upon different treatments (Supplemental Figure 2G), inferring a key role of LGG in upregulating IL-10 expression in monocytes during DSS-induced colitis. To determine this, we conducted qPCR experiments with Ly6C^+^ monocytes isolated from MLNs. The transcriptional level of *Il10* was significantly increased in monocytes from LGG+DSS-treated mice compared with those from DSS-treated mice (*P* = 0.0001; Figure 2F). Consistently, at the protein level, we observed an increase of IL-10 in these specific monocytes from MLNs upon LGG+DSS treatment, evidenced by the ELISA assay (Figure 2G). Of note, LGG alone had little effect on the expression of IL-10 in vivo or ex vivo (Figure 2, A, B, F, and G). We thus reasoned that LGG induction of IL-10 in monocytes was specific to colitis or select inflammatory stress. To verify this, we isolated Ly6C^+^ monocytes from non-DSS- and DSS-primed mice and treated them with live LGG ex vivo. LGG upregulated IL-10 only in DSS-primed monocytes and was unable to trigger IL-10 expression in non-DSS-primed monocytes or bone marrow monocytes in vitro (Figure 2H and Supplemental Figure 2H). Collectively, our data indicate that LGG licenses IL-10 accumulation in monocytes upon the DSS-induced inflammation.

### LGG suppresses colitis relying on STING signaling in Ly6C^+^ monocytes.

**Figure 3:**
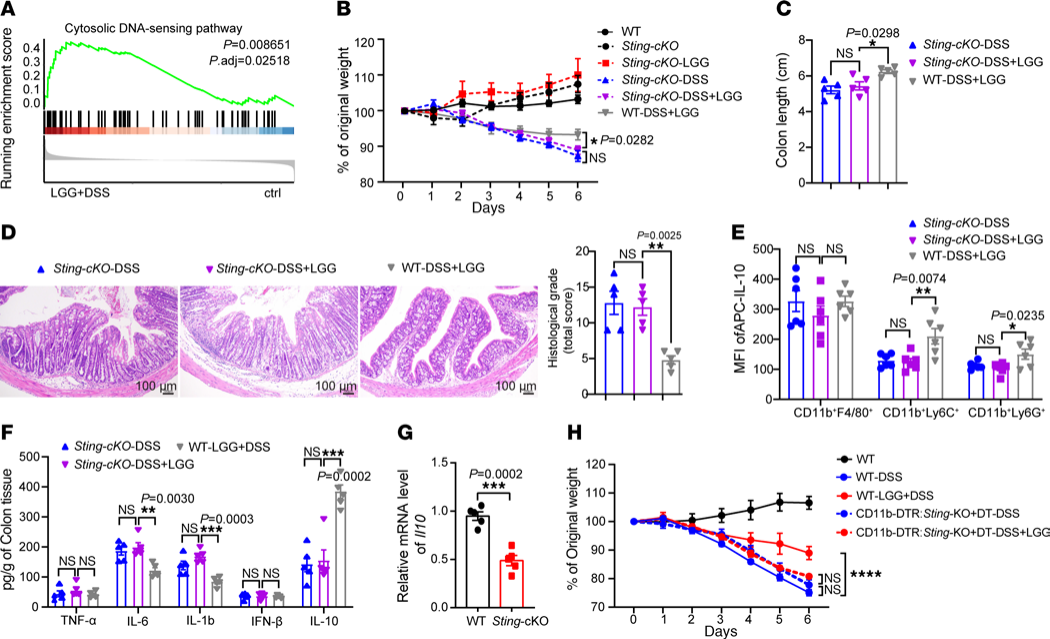
LGG restrains DSS-induced colitis in a STING-dependent manner. (**A**) Gene set enrichment analysis plots of the cytosolic DNA-sensing pathway were positively correlated with LGG+DSS treatment. (**B**) Body weight assessment of *Lyz*^cre^*Sting*^fl/fl^ conditional KO mice that received 3% DSS in drinking water for 7 days. Mice were orally administered LGG daily for 2 weeks (*n* = 5 per group). (**C**) Colon length of *Sting*-cKO mice with DSS or LGG+DSS treatment and WT mice with LGG+DSS treatment on day 7 (*n* = 5 per group). (**D**) Representative images of H&E-stained colon tissues and the matching histological grade score (*n* = 5 per group) in mice with different treatments (day 7). Scale bar: 100 μm. (**E**) Mean fluorescence intensity (MFI) of IL-10 in different myeloid cells in MLNs from *Sting*-KO or WT mice with the indicated treatment (*n* = 6 per group). (**F**) Colon tissues were collected from different mice as indicated in **B** and used to detect the protein levels of inflammatory cytokines (*n* = 5 per group). (**G**) Ly6C^+^ monocytes were isolated from DSS-treated WT and *Sting*-cKO mice and cocultured with LGG prior to *Il10* mRNA qPCR analysis (*n* = 5 per group). (**H**) CD11b-DTR:*Sting*-KO bone marrow chimeric mice were injected with diphtheria toxin (DT, 100 ng/per mouse). The next day, mice were treated with 3% DSS treatment for 7 days. Mice were orally administered LGG daily for 2 weeks (*n* = 5 per group). Data are expressed as mean ± SEM. One of 2 or 3 representative experiments is shown. Statistical analysis was performed using 2-way ANOVA test with corrections for multiple variables (**B** and **H**), 1-way ANOVA with Bonferroni’s multiple comparison tests (**C**–**F**), and unpaired 2-tailed Student’s *t* tests (**G**) *P < 0.05, **P < 0.01, ***P < 0.001, ****P < 0.0001.

STING has been implicated in inflammatory colitis via diverse mechanisms (19, 23), and we found via our RNA-Seq analyses of monocytes isolated from MLNs of LGG+DSS-treated WT mice that the “Cytosolic DNA-sensing pathway” was activated in LGG+DSS-treated monocytes (Figure 3A). We therefore aimed to delineate the functional role of STING in LGG-mediated protective activity using a myeloid-specific conditional KO mouse model, *Lyz*^Cre+^*Sting*^fl/fl^ (hereafter referred to as *Sting*-cKO), and *Sting*^fl/fl^ (hereafter WT) mice for the aforementioned in vivo and ex vivo experiments. STING depletion in myeloid cells elicited weight lost in response to DSS treatment over a 7-day period (Figure 3B); supplementation of LGG to these mice failed to reverse the weight loss and colon length shortening (*P* = 0.6531 and *P* = 0.8597, respectively; Figure 3, B and C). Similarly, there was no improvement in histological damage in *Sting*-cKO mice following LGG treatment (Figure 3D). Our data indicate that myeloid cell–specific STING plays a vital role in the protective activity of live LGG against DSS-induced colitis.

To explore whether IL-10 is under the control of STING signaling in myeloid cells in our system, we measured the level of IL-10 in various myeloid cell compartments in MLNs from *Sting*-cKO mice. Flow cytometry analysis showed that there were not significant changes of IL-10 expression in any monocyte population (CD11b^+^F4/80^+^, CD11b^+^Ly6C^+^, and CD11b^+^Ly6G^+^) or other immune cell types with LGG+DSS versus DSS treatment alone (Figure 3E and Supplemental Figure 3A). To further verify this, the colon tissues were collected 7 days after DSS treatment and were subjected to ELISA and qPCR analyses. As expected, LGG treatment did not affect the IL-10 level in colon tissue at both the mRNA and protein levels in *Sting*-cKO mice (Figure 3F and Supplemental Figure 3B). Correspondingly, the levels of proinflammatory cytokines (TNF-α, IL-6, IL-1b, and IFN-β) in colon tissues were unaffected with LGG treatment (Figure 3F and Supplemental Figure 3B), plausibly due to the unchanged IL-10. Collectively, our observations suggest that STING signaling is required to generate the antiinflammatory cytokine IL-10 in myeloid cells after LGG administration and plays a role in maintaining gut immune homeostasis in colitis.

Having demonstrated that CD11b^+^Ly6C^+^ monocytes were seen to be required to mediate the protective effects of LGG against colitis, we sought to further investigate whether STING signaling in CD11b^+^ monocytes is similarly required. We treated the isolated monocytes from DSS-treated WT and *Sting*-cKO mice with LGG ex vivo and measured their IL-10 levels. qPCR analysis showed that STING deficiency significantly decreased IL-10 mRNA expression in CD11b^+^ monocytes (Figure 3G). Next, we reconstituted irradiated mice with a mixture of *Sting*-KO bone marrow cells (BMCs) and WT BMCs from CD11b–diphtheria toxin receptor (CD11b-DTR) mice (at a ratio of 1:1). Upon administration of diphtheria toxin, WT monocytes expressing CD11b-DTR were selectively eliminated, with all remaining monocytes being *Sting* deficient. We found that DSS treatment induced body weight loss at a similar level in diphtheria toxin–treated CD11b-DTR:*Sting*-KO chimeric mice and WT mice (Figure 3H). Notably, oral administration of live LGG was sufficient to rescue weight loss and colon shortening in DSS-treated WT mice (WT-LGG+DSS vs. WT-DSS, *P* < 0.0001; Figure 3H and Supplemental Figure 3C) but not in DSS-treated CD11b-DTR:*Sting*-KO chimeric mice with diphtheria toxin treatment (CD11b-DTR:*Sting*-KO+DT-DSS+LGG vs. CD11b-DTR:*Sting*-KO+DT-DSS, not significant; Figure 3H and Supplemental Figure 3C). These data demonstrate that monocyte-specific *Sting* depletion impairs the ability of LGG to alleviate colitis. It is thus conceivable that STING does contribute to the regulation of IL-10 expression in monocytes during colitis.

### LGG induces IL-10 expression in monocytes via the STING/TBK1/RELA axis.

**Figure 4:**
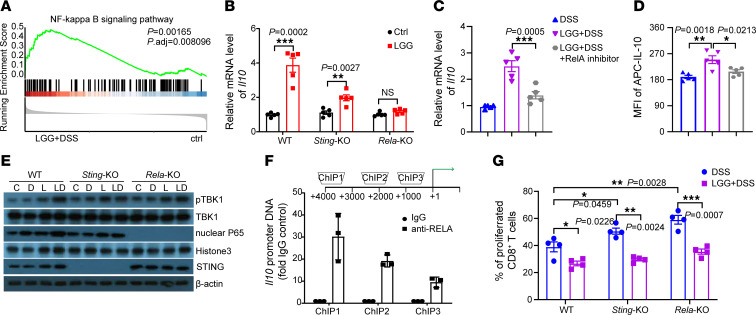
LGG induces IL-10 expression in monocytes via the STING/RELA axis during colitis. (**A**) Gene set enrichment analysis plots of the NF-κB signaling pathway were positively correlated with LGG+DSS treatment based on the RNA-Seq data of CD11b^+^ monocytes isolated from MLNs. (**B**) qPCR analysis of *Il10* in monocytes isolated from DSS-treated WT, *Sting*-KO (*Tmem173*^−/−^ whole-body KO), and *Rela*-KO (*Lyz*^cre^*Rela*^fl/fl^ conditional KO) mice with or without LGG treatment (*n* = 5 per group). (**C** and **D**) Monocytes were isolated from DSS-treated WT mice and cocultured with live LGG with or without RelA inhibitor (sulfasalazine, 5 mM). The mRNA (**C**) and protein levels (**D**) were measured by qPCR analysis and flow cytometry analysis, respectively (*n* = 5 per group). (**E**) Ly6C^+^ monocytes were collected from the MLNs of WT, *Sting*-KO, and *Rela*-KO mice with DSS with or without LGG treatment. (**F**) ChIP qPCR analysis of the *Il10* promoter in the collected monocytes from LGG+DSS-treated WT mice (*n* = 3 per group). (**G**) Flow cytometry analysis of an in vitro proliferation assay showing the frequency of proliferating CD8^+^ T cells when cocultured with monocytes as indicated in **C** (*n* = 4 per group). Data are expressed as mean ± SEM. One of 2 or 3 representative experiments is shown. Statistical analysis was performed using 1-way ANOVA with Bonferroni’s multiple comparison tests (**C**, **D**, and **G**) and unpaired 2-tailed Student’s *t* tests (**B**). *P < 0.05, **P < 0.01, ***P < 0.001.

To investigate the molecular mechanism underlying STING-driven IL-10 production, we reanalyzed our RNA-Seq data of monocytes isolated from MLNs of LGG+DSS-treated WT mice. Gene set enrichment analysis identified that the gene signature for the “NF-κB signaling pathway” was strongly skewed toward the LGG+DSS treatment (Figure 4A). Given that the NF-κB pathway is downstream of cGAS/STING signaling, we hypothesized that LGG may induce IL-10 expression in a STING/NF-κB-dependent manner in the context of colitis. To examine this, we conducted a series of qPCR experiments with monocytes isolated from DSS-treated WT, *Sting*-KO (*Tmem173*^−/−^ whole-body KO) and *Rela*-KO (*Lyz*^cre^*Rela*^fl/fl^ conditional KO, a canonical downstream mediator of NF-κB pathway) mice. The results showed that LGG treatment strongly upregulated *Il10* mRNA expression in WT monocytes, while *Il10* expression in *Sting*-deficient or *RelA*-deficient monocytes was reduced (Figure 4B). Moreover, the RelA inhibitor (sulfasalazine) abolished *Il10* induction by LGG treatment in DSS-primed WT monocytes (Figure 4C). We also observed consistent trends in IL-10 levels in monocytes by flow cytometry (Figure 4D). In addition, we collected Ly6C^+^ monocytes from WT, *Sting*-KO, and *Rela*-KO mice with DSS with or without LGG treatment to detect the phosphorylation level of molecules involved in STING/RELA signaling. The level of TBK1 phosphorylation was notably increased in WT and *Rela*-KO monocytes upon LGG+DSS treatment but compromised in *Sting*-deficient monocytes (Figure 4E). We also observed an increase in nuclear RELA in WT monocytes that appeared to stay unchanged in *Sting*-KO monocytes after LGG+DSS treatment (Figure 4E).

Next, to further confirm the key role of RELA in IL-10 regulation in monocytes, we analyzed a large-scale ChIP sequencing dataset consisting of mouse macrophages after lipid A treatment (24). We found consistent evidence that the promoter region of IL-10 harbored sites for RELA (Supplemental Figure 4A). Then, we performed an anti-RELA antibody-based ChIP assay with monocytes isolated from LGG+DSS-treated WT mice. ChIP-qPCR revealed that RELA interacted directly with the IL-10 promoter region (Figure 4F). Consistent with the immunosuppressive function of STING- and RELA-driven IL-10, monocyte-specific KO of either *Sting* or *RelA* led to a decreased ability to suppress CD8^+^ T cell proliferation but was rescued, at least partially, in the presence of LGG (Figure 4G). Furthermore, we did not observe the activation of IRF3, a canonical downstream signal of STING, upon LGG or LGG+DSS treatment (Supplemental Figure 4B). Collectively, these findings indicate that STING, TBK1, and RELA are critical components of IL-10 activation by LGG in the context of colitis.

### LGG triggers an IL-10–based autocrine regulatory loop in monocytes during colitis.

**Figure 5:**
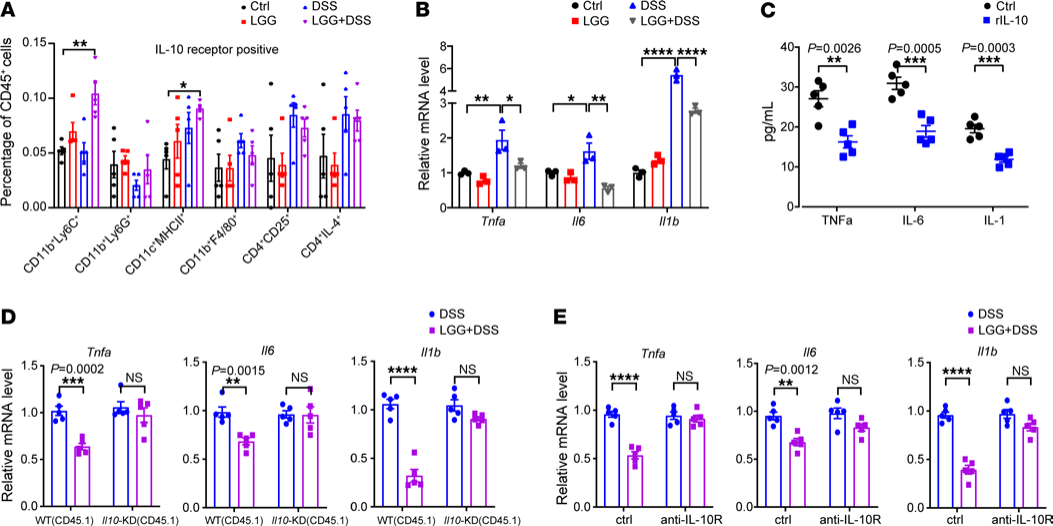
LGG triggers an IL-10–based autocrine regulatory loop in monocytes during colitis. (**A**) Percentages of IL-10R^+^ immune cells in MLNs from WT mice with the indicated treatment (*n* = 5 per group). (**B**) Ly6C^+^ monocytes were isolated from MLNs in WT mice with different treatments as indicated and subjected to qPCR analysis of the mRNA levels of *Tnfa*, *Il6*, and *Il1b* (*n* = 3 per group). (**C**) Ly6C^+^ monocytes were isolated from LGG+DSS-treated mice and stimulated with recombinant IL-10 (10 ng/mL) for 60 minutes. The supernatant was collected to detect the protein levels of inflammatory cytokines using a LEGENDplex cytokine kit (*n* = 5 per group). (**D**) CD45.1 monocytes (WT or *Il10r* knockdown) were sorted after coculture with CD45.2 monocytes from MLNs of DSS- or LGG+DSS-treated mice and subjected to qPCR analysis of the mRNA levels of *Tnfa*, *Il6*, and *Il1b* (*n* = 5 per group). (**E**) qPCR analysis of monocytes (as indicated in **D**) in the presence of a neutralizing anti–IL-10R antibody (10 μg/mL) (*n* = 5 per group). Data are expressed as mean ± SEM. One of 2 or 3 representative experiments is shown. Statistical analysis was performed using 1-way ANOVA with Bonferroni’s multiple comparison tests (**A** and **B**) and unpaired 2-tailed Student’s *t* tests (**C**–**E**). *P < 0.05, **P < 0.01, ***P < 0.001, ****P < 0.0001.

To investigate the physiological role of LGG-induced IL-10 and its effector cells in gut, we profiled the distribution of the IL-10 receptor (IL-10R) among different immune cells isolated from the MLNs in DSS-induced colitis mice with LGG treatment. CD11b^+^Ly6C^+^ monocytes made up a sizable portion of IL-10R^+^ cells after treatment with LGG+DSS, as measured by both flow cytometry and qPCR (Figure 5A and Supplemental Figure 5A). We also observed an increase in the “Cytokine-cytokine receptor interaction” pathway based on RNA-Seq of monocytes isolated from LGG+DSS-treated mice, comprising in part both *Il10* and *Il10rb* expression (Supplemental Figure 5B). We thus reasoned that IL-10 produced by monocytes could activate their own IL-10 signaling pathway, which has been proved to reduce the production of proinflammatory cytokines (25), resulting in an autocrine signaling axis. To this end, we collected Ly6C^+^ monocytes from MLNs for the qPCR analyses. The results demonstrated a huge increase of TNF-α, IL-6, and IL-1b in Ly6C^+^ monocytes from DSS-treated mice and a decrease in LGG+DSS-treated mice compared with those in DSS-treated mice (Figure 5B). Furthermore, stimulation with recombinant IL-10 decreased expression of TNF-α, IL-6, and IL-1b in Ly6C^+^ monocytes isolated from LGG+DSS-treated mice (Figure 5C). These data suggest that Ly6C^+^ monocytes are established major producers of intestinal IL-10 that regulate expression of multiple inflammatory cytokines in colitis.

We next employed an in vitro coculture system to probe for the existence of a monocyte-based IL-10 autocrine regulatory loop. Bone marrow–derived *Il10r*-knockdown monocytes (CD45.1 *Il10r*-KD) were generated using *Il10r-*siRNA (Supplemental Figure 5C) and cultured with monocytes isolated from the MLNs of LGG+DSS-treated mice (CD45.2). After culturing, CD45.1 monocytes were sorted by flow cytometry, and the levels of proinflammatory cytokines were examined. As expected, mRNA levels of TNF-α, IL-6, and IL-1b were significantly decreased in WT monocytes but not in *Il10r*-KD monocytes (Figure 5D). Treatment with a neutralizing anti–IL-10R antibody elicited a comparable phenotype (Figure 5E). Similarly, recombinant IL-10 treatment failed to trigger the production of TNF-α, IL-6, and IL-1b in *Il10r*-KD monocytes (Supplemental Figure 5D). Together, these observations suggest that monocytes are both the main producer of intestinal IL-10 and simultaneously sense this cytokine by virtue of their IL-10R expression, supporting the previous notion that the IL-10/IL-10R axis plays a key role in suppressing the expression of proinflammatory cytokines. Collectively, our results demonstrate that LGG triggers an IL-10–based autocrine regulatory loop in monocytes to suppress DSS-induced colitis.

### LGG shapes the gut microbial community and metabolic function associated with intestinal immune responses.

**Figure 6:**
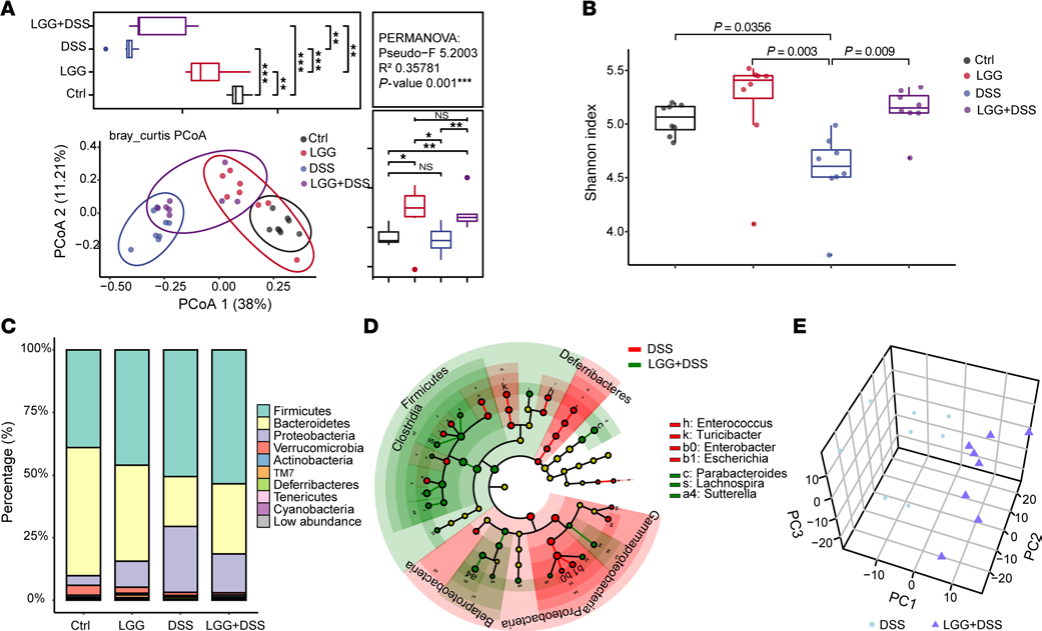
LGG shapes the gut microbial community and its metabolic function associated with intestinal immune responses. (**A**) Unconstrained principal coordinate analysis (PCoA) with Bray-Curtis distance indicates microbial community differences (variation) across treatments. The distribution of the first two most informative coordinates was performed in the corresponding marginal box plot. Each data point corresponds to a sample, which is colored according to the treatment. Ellipses represent a 95% CI around the cluster centroid. PERMANOVA detected significant differences (*P* < 0.05) among different treatments, and all pairwise differences were statistically significant with *P* values adjusted using the Benjamini and Hochberg methods. (**B**) Box plot of the Shannon index represents α diversity for intestinal microbiota from the indicated treatment groups. Statistical significance was assessed using ANOVA and *t* test. All *P* values were adjusted using the Benjamini and Hochberg methods. (**C**) Distribution of the intestinal microbiota at the phylum level. Taxonomic composition distribution was labeled with different colors; the top 9 phyla ranked by the average counts among all samples are illustrated. “Low abundance” represents the combination of the rest of the phyla. (**D**) Cladogram showing differential bacterial abundance between DSS (red) and LGG+DSS (green) treatment groups based on LEfSe analysis. The levels indicate, from the inner to outer rings, phylum, class, order, family, and genus. Linear discriminant analysis score for discriminative features >3. (**E**) Score plot of the first 3 principal components analysis calculated on fecal metabolites. Each sphere represents 1 LC-MS sample. Spheres are colored according to treatment group: the DSS group is in blue and the LGG+DSS group is in purple. DSS fecal samples and LGG+DSS fecal samples separated from each other, indicating that the metabolome profiles were different. The variance explained by each PC is reported. The 3 major principal components explained the percentage of the cumulative variance. *P < 0.05, **P < 0.01, ***P < 0.001, ****P < 0.0001.

LGG exhibited more pronounced protective activity against DSS-induce colitis in GF mice than SPF mice. A growing body of evidence suggests a correlation between commensal dysbiosis and intestinal inflammation (26). These made us hypothesize that changes in the gut microbiome mediate the protective effects of LGG. To profile the gut microbiota underlying the inoculation of LGG in mice especially during DSS-induced colitis, we performed bacterial 16S rRNA sequencing, targeting the V3–V4 region, of colon content samples from mice treated with DSS and/or LGG. Principal coordinate analysis (PCoA) based on the distance matrix of Bray-Curtis dissimilarity revealed significant changes in the overall structure of colonic microbiome across different treatments (permutational multivariate analysis of variance [PERMANOVA]/Adonis test, *P* = 0.001; Figure 6A). Gut microbial diversity, as determined by both Shannon diversity (Figure 6B) and the Simpson index estimation (Supplemental Figure 6A), was significantly reduced in DSS-treated mice compared with untreated control mice (*P* < 0.05). Of note, the diversity of the gut microbiota in LGG+DSS-treated mice remained comparable with that of control mice (Figure 6B and Supplemental Figure 6A), suggesting that LGG reverts the aberrant microbiota composition caused by DSS.

To elucidate which bacteria were the most important determinants in shifting the overall structure of gut microbiota, we further analyzed the taxonomic composition of the gut microbiome upon different treatments. We found that DSS-treated mice displayed a higher abundance of *Proteobacteria* and *Deferribacteres* and a lower abundance of *Bacteroidetes* and *Saccharibacteria* in comparison with control mice (Figure 6C and Supplemental Figure 6B). As expected, treatment with LGG in DSS-treated mice tended to reverse this population shift (Figure 6C and Supplemental Figure 6B). We also identified enriched amplicon sequence variants according to their taxonomy using Manhattan plots. In particularly, *Bacteroidetes* was enriched in LGG+DSS-treated mice compared with DSS-treated mice (Supplemental Figure 6C) and has previously been implicated in protection from DSS-induced intestinal inflammation (27, 28). To identify the specific bacterial taxa and predominant bacteria upon LGG+DSS treatment that accounts for the greatest differences with and without LGG inoculation, we performed linear discriminant analysis effect size (LefSe). *Lachnospira*, *Parabacteroides*, and *Sutterella* (in genera) were enriched in LGG+DSS-treated mice (Figure 6D). These bacteria are known to contribute to the maintenance and integrity of the intestinal epithelium, thereby reducing colonic tissue damage and inflammation (29–31). In contrast, *Enterobacter*, *Enterococcus*, *Turicibacter*, and *Escherichia*, which are known to play a proinflammatory role (32, 33), were enriched in DSS-treated mice (Figure 6D).

To verify the contribution of the LGG-changed fecal microbiome to the observed effects to rescue DSS-induced colitis, we employed GF mice to conduct the FMT experiment using fresh feces from WT SPF mice without any treatment or DSS-treated WT SPF mice and treated these mice with orally administration of LGG. LGG treatment resulted in a significant improvement in body weight loss induced by FMT from DSS-treated mice (Supplemental Figure 6D). Furthermore, we used GF-*Il10*-KO mice for the aforementioned FMT experiments. We inoculated fresh feces from SPF WT mice into GF-*Il10*-KO mice and found that these mice exhibited more severe weight loss compared with control mice after 7 weeks (Supplemental Figure 6E). We next performed the FMT experiment with fresh feces from LGG-training mice transplanted into GF *Il10*-KO (GF-*Il10*-KO-LGG-FMT) mice over 7 weeks, and we observed a significant improvement in weight loss compared with that of GF-*Il10*-KO-WTSPF-FMT mice (Supplemental Figure 6E). Of note, GF-*Il10*-KO-LGG-FMT mice were unable to gain weight to the level of GF-*Il10*-KO control mice or SPF-WT-LGG-FMT mice given antibiotics (FMT was performed with fresh feces from LGG-training mice transplanted into antibiotic cocktail [ABX]-treated WT mice) (Supplemental Figure 6E). Taken together, LGG-mediated antiinflammatory effects require not only the IL-10 production in monocytes but also the changed gut microbial composition.

In order to examine the effects of the LGG-reshaped microbiome on the predicted metabolic function during colitis, we utilized PICRUSt software based on the KEGG database using 16S RNA-Seq data. Essential and branched-chain amino acid biosynthesis and energy metabolism were all enriched in the prediction among DSS+LGG-treated groups (Supplemental Figure 7A). These metabolic functions have previously been shown to contribute to the maintenance of intestinal homeostasis and immune responses (34, 35). We next profiled colonic metabolites using an untargeted metabolomics approach. Partial least-squares discriminant analysis (Figure 6E) and hierarchical clustering analysis of the top 75 differential metabolites (Supplemental Figure 7B) revealed big changes after LGG administration in DSS-induced colitis. Differential metabolites were identified by volcano plot analyses (Supplemental Figure 7C). LGG significantly increased pathways involved in energy metabolic homeostasis, including quinone biosynthesis and retinol metabolism (Supplemental Figure 7D). These observations led us to hypothesize that LGG altered the metabolome by reshaping the gut microbiome. Consistent with this, we observed tight connections using an interomics correlation analysis of abundances in colonic 16s sequencing (Supplemental Figure 7E). Collectively, our findings suggest that LGG modulates the abundance of specific commensal bacteria and metabolic function associated with intestinal immune responses.

### FMT from LGG-colonized mice alleviates immune checkpoint blockade–associated colitis.

**Figure 7:**
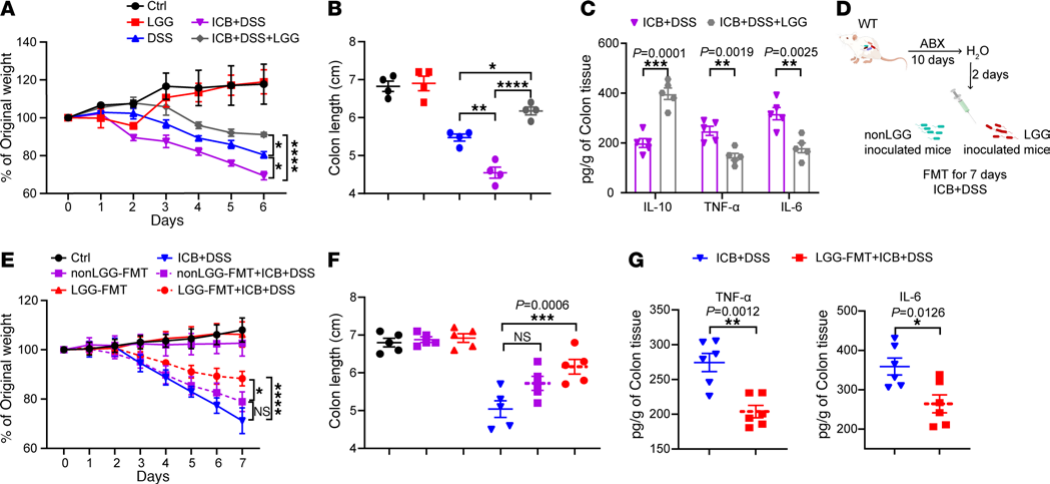
Oral administration of live LGG prevents immune checkpoint blockade–associated colitis. (**A**) Body weight assessment of WT mice treated with 3% DSS (for 7 days), ICB (ICB: 100 μg anti–CTLA-4 mAb and 250 μg anti–PD-1 mAb), and/or oral administration of live LGG (*n* = 5 per group). (**B**) Colon length of mice as indicated in **A** (*n* = 5 per group). (**C**) The protein levels of IL-10, TNF-α, and IL-6 in colon tissues from the mice, as indicated in **A**, assessed using a LEGENDplex cytokine kit (*n* = 5 per group). (**D**) Schematic image illustrating FMT experimental design. WT mice were treated with broad-spectrum antibiotics for 10 days followed by regular water for 2 days. FMT was performed with fresh feces from non-LGG or LGG-training mice into ABX-treated WT mice donor mice via oral gavage every other day for 7 days with or without ICB+DSS treatment. (**E** and **F**) Body weight change (**E**) and colon length (**F**) of the indicated mice and treatments (*n* = 5 per group). (**G**) The protein levels of TNF-α and IL-6 in colon tissues from the indicated mice, assessed using a LEGENDplex cytokine kit (*n* = 6 per group). Data are expressed as mean ± SEM. One of 2 representative experiments is shown. Statistical analysis was performed using 2-way ANOVA test with corrections for multiple variables (**A** and **E**), 1-way ANOVA with Bonferroni’s multiple comparison tests (**B** and **F**), and unpaired 2-tailed Student’s *t* tests (**C** and **G**). *P < 0.05, **P < 0.01, ***P < 0.001, ****P < 0.0001.

Immune checkpoint blockade (ICB) has revolutionized the treatment of cancer and other diseases (36, 37) but with limited usage due to the immune-related adverse effects, including ICB-associated colitis (38, 39). ICB-associated colitis shares several features with IBD pathophysiology (40). We tested the therapeutic potential of LGG to suppress ICB-associated colitis in mice treated with a combination of anti–CTLA-4 and anti–PD-1 antibodies in addition to DSS. Mice receiving ICB treatment exhibited more severe weight loss and colon length shortening compared with mice receiving DSS treatment alone (Figure 7, A and B), consistent with previous studies that identified ICB worsened DSS-induced colitis (41). Oral gavage of live LGG significantly rescued the severe weight loss and colon length shortening induced by ICB treatment in the DSS-induced colitis mice (Figure 7, A and B). Similarly, LGG also increased IL-10 and decreased two proinflammatory cytokines (TNF-α and IL-6) in colon tissues in mice treated with ICB+DSS (Figure 7C). Our findings indicate that oral administration of live LGG can alleviate ICB-associated colitis.

There is an urgent clinical need for prophylactic measures to prevent ICB-associated colitis. We hypothesized that FMT from LGG-colonized mice could prevent ICB-associated colitis (Figure 7D). FMT from LGG-inoculated mice administered to ICB+DSS-induced colitis mice for 7 days resulted in a significant improvement in weight loss and colon length (Figure 7, E and F). Moreover, FMT significantly reduced the expression of TNF-α and IL-6 in colon tissues in ICB+DSS-induced colitis mice (Figure 7G). Our results are consistent with a case report in which refractory ICB-associated colitis was successfully managed with FMT (42) and add to the growing body of evidence that gut microbiome modulation can alleviate ICB-associated colitis.

## Discussion

Our study establishes an empirical basis for developing oral administration of live LGG probiotics for preventing inflammatory colitis. We demonstrate that the STING/RELA axis is required for IL-10 production in monocytes with LGG treatment in colitis. Furthermore, LGG treatment is associated with gut microbiota and their metabolic function that likely contribute to its protective activity against colitis. Our findings offer valuable insight into the molecular mechanism of an LGG-mediated antiinflammatory role of monocyte-STING in inflammatory colitis and highlight its potential to prevent colitis.

It has been reported that several myeloid cell populations can produce IL-10 in different inflammatory conditions. For instance, macrophages produce IL-10 after LPS stimulation and in a mouse model of colitis (43). Intestinal CX3CR1^hi^CD11b^+^CD11c^+^ cells suppress intestinal inflammation in an IL-10–dependent manner (44), and Ly6C^–^ monocytes are a major IL-10 producer in inflammatory liver infection (45). In our case, we provided evidence that Ly6C^+^ monocytes in the gut are the major source of LGG-induced IL-10 during inflammatory colitis. Of note, we also observed increased IL-10 production in Ly6G^+^ monocytes after LGG+DSS treatment. Hence, we cannot rule out an additional role for other myeloid subsets in LGG-induced IL-10 production during colitis. More importantly, our findings demonstrate that IL-10 production by an immune cell population (Ly6C^+^ monocytes) is sufficient to prevent colitis. Interestingly, LGG induction of monocytic IL-10 was specific to colitis, and LGG had little effect on IL-10 expression in mice without DSS treatment.

Both STING activation and deficiency have been separately linked to the exacerbation of DSS-induced colitis (15, 46). LGG can also induce proinflammatory cytokine expression in intestinal DCs via STING activation (11). We observed that LGG activation of STING in Ly6C^+^ monocytes promotes expression of the antiinflammatory cytokine IL-10 in this study. Thus, it is likely that the downstream mediators of STING activation are immune cell specific, allowing them to maintain homeostasis in the presence of various stresses. To the best of our knowledge, this is the first report on the involvement of STING signaling in antiinflammatory immune responses against colitis. We also identified RELA as a mediator of the STING/IL-10 axis; however, we are not able to definitively exclude the possible involvement of other mediators, such as STAT1/3, JUN, and GATA3, all of which have been shown to contribute to IL-10 regulation in immune cells (47, 48). To discern the STING-mediated antiinflammatory mechanism, it is essential to clarify which mediator is required for responding different stresses.

There is increasingly growing interest on the role of the gut microbiome and metabolic activity in intestinal inflammation (49). In this study, we provide the evidence that LGG normalizes the composition of the gut microbial community and influences its metabolic activities, which may be critical for the prevention of colitis. These shifting trends are consistent with those in patients with IBD, in which dysbiosis is characterized by an increase in proinflammatory bacteria (mostly *Proteobacteria*) and a decrease in commensal beneficial bacteria (mostly *Firmicutes*) (50, 51). In addition, it remains possible that the LGG modulated microbiota might be beneficial for IL-10–dependent antiinflammatory immunity. Several lines of evidence support a critical role for IL-10–associated microbiota in the experimental colitis system. Intragastric administration of IL10-producing *Lactococcus lactis* also protects mice from DSS-induced colitis and can prevent the development of spontaneous colitis in IL10-deficient mice (52), and *Helicobacter hepaticus* and *Bacteroides fragilis* polysaccharides can induce IL-10 production from gut-resident MNPs and T cells (53, 54). Thus, using IL-10–associated probiotics may be a more rational and effective strategy to prevent inflammatory colitis.

In the clinical setting, recombinant IL-10 treatment or intestinal delivery of IL-10 did not result in significant clinical benefits in patients with IBD, with unclear side effects (55). We here provide proof-of-principle preclinical evidence that oral administration of live LGG or FMT of LGG stool ameliorates DSS-induced colitis, signifying that LGG might be a more efficient and safer strategy to prevent colitis, due to the fact that LGG simultaneously modulates IL-10 expression and reshapes the gut microbiota. The clinical importance of these observations is significant. Oral administration of live LGG could be also an ideal and safe strategy for the treatment of other types of inflammation-associated diseases.

## Methods

### Sex as a biological variable.

We used female mice of 6–8 weeks of age for all experiments. Our study exclusively examined female mice. It is unknown whether the findings are relevant for male mice.

### In vivo animal studies.

All mice were housed and used according to the animal experimental guidelines set by the Animal Care and Use Committee of the Institute of Animal Science, Chinese Academy of Agricultural Sciences. All animals were maintained in pathogen-free conditions and cared for in accordance with the International Association for Assessment and Accreditation of Laboratory Animal Care policies and certification.

For generation of inflammation mouse models, C57B6/J mice were treated with 3% DSS in drinking water for 7 days. Control C57B6/J mice were given regular drinking water. For ICB-associated colitis, the mice were injected once every other day (initiated 3 days before the 3% DSS administration) with 100 μg anti–CTLA-4 antibody (Bioxcell; catalog BE0131; clone 9H10) and 250 μg anti–PD-1 antibody (Bioxcell; catalog BE0146; clone RMP1-14) or isotype control antibody before the DSS administration. For bacteria administration, LGG was cultured in MRS medium at 37°C for 24–36 hours, and the selected mice were orally gavaged with 2 × 10^9^ CFU LGG. For adaptive transfer experiments, we treated WT and *Il10*-KD BM monocytes with live LGG in vitro and used them in the following adaptive transfer experiments, 5 × 10^6^ cells (per mice) were injected i.v. into recipient mice.

### Flow cytometry.

For flow cytometric analysis, MLNs were collected from mice and were then used to generate single-cell suspensions by grinding the tissues through 70 μm filters. Samples were then filtered through a 70 μm cell strainer and washed twice with staining buffer. The cells were resuspended in staining buffer and were blocked with anti-FcR (BioXcell; catalog BE0307; clone 2.4G2). Subsequently, the cells were stained with antibodies for 15–30 minutes at 4°C in the dark and then detected by flow cytometry with a BD Fortessa. For intracellular staining, cells were first permeabilized using a Fixation and Permeabilization Kit (BD) and then stained with appropriate antibodies. Analysis of flow cytometry data was performed using FlowJo (version 10, BD).

### Cytokine detection.

Colon tissues or MLNs were homogenized in PBS with protease inhibitor and then centrifuged at 13,400 g for 10 minutes to collect the supernatant. The supernatant was used to detect the cytokines with the LEGENDplex Mouse Inflammation Panel (13-plex) with V-bottom Plate kit (BioLegend). The samples were detected by flow cytometry with a BD Fortessa. The obtained flow data was analyzed with LEGENDplex software (v8.0, BioLegend).

### Generation of bone marrow chimeras.

WT mice were irradiated with a single dose of 8 Gy. The irradiated mice were adoptively transferred (i.v.) with 2.5 × 10^6^ *Sting*-KO BMCs and WT BMCs from CD11b-DTR mice (at a ratio of 1:1). The mice were treated with neomycin (0.5 mg/mL) diluted in drinking water for 4 weeks after reconstitution and then were used for further study.

### CD8^+^ T cell suppression assay.

Ly6C^+^ monocytes were isolated from MLNs in DSS or LGG+DSS-treated WT, *Sting*-KO, and *Rela*-cKO mice and were used to perform the suppression assay. CD8^+^ T cells were isolated from the spleens of naive mice by using the EasySep Mouse CD8^+^ T Cell Isolation Kit (STEMCELL) according to manufacturer’s instructions and then stained with CellTrace CFSE (Invitrogen). The CD8^+^ T cells were cultured with anti-CD3/anti-CD28 beads and were cocultured with monocytes at a ratio of 4:1. The CD8^+^ T cell proliferation was analyzed by flow cytometry.

### FMT.

WT mice were treated with broad-spectrum antibiotics for 10 days followed by regular water for 2 days. FMT was performed with fresh feces from non-LGG or LGG-training mice into ABX-treated WT mice donor mice via oral gavage every other day. Thereafter, mouse FMT was performed with or without the presence of 3% DSS and ICB for a period of 7 days.

### Histopathology and inflammatory score in colitis model.

Formalin-fixed mice colon tissues were processed and stained with H&E according to standard procedures. The stained intestinal sections were graded by a blinded scorer. The colon tissues were scored on a 0–4 system: 0, normal; 1, mild inflammation with less than 10% loss of epithelial structure (crypts) and focal enterocyte hyperplasia; 2, moderate inflammation with 10%–30% of crypt loss, multifocal enterocyte hyperplasia, and goblet cell loss; 3, obvious inflammation with 30%–50% of crypt loss, diffuse enterocyte hyperplasia, and few goblet cells; 4, severe inflammation with over 50% of crypt loss, diffuse enterocyte hyperplasia, and mucosal ulceration.

### Reagents and antibodies.

The following reagents and antibodies were purchased from BioLegend and used for flow cytometry: APC/Cy7 anti-mouse CD45 antibody (catalog 147717; clone I3/2.3), BV421 anti-mouse CD11b antibody (catalog 101235; clone M1/70), PE anti-mouse F4/80 antibody (catalog 111603; clone W20065B), APC anti-mouse IL-10 antibody (catalog 505009; clone JES5-16E3), FITC anti-mouse Ly6C antibody (catalog 128005; clone HK1.4), AF700 anti-mouse Ly6G antibody (catalog 127621; clone 1A8), BV421 anti-mouse CD11c antibody (catalog 117329; clone N418), AF700 anti-mouse MHC II antibody (catalog 107621; clone M5/114.15.2), PE anti-mouse IL-10 R antibody (catalog 112705; clone 1B1.3a), Pacific blue anti-mouse CD45 antibody (catalog 157212; clone S18009F), APC anti-mouse CD4 antibody (catalog 100411; clone GK1.5), FITC anti-mouse CD25 antibody (catalog 101907; clone 3C7), and BV711 anti-mouse IL-4 antibody (catalog 504133; clone 11B11).

InVivoMAb anti-mouse IL-10 (catalog BE0049; clone JES5-2A5) was purchased from BioXcell for use in mouse studies. The following reagents and antibodies were purchased from Cell Signaling Technology and used for immunoblotting: TBK1/NAK (D1B4) Rabbit mAb (catalog 3504), Phospho-TBK1/NAK (Ser172) (D52C2) (catalog 5483), NF-κB p65 (D14E12) Rabbit mAb (catalog 8242), STING (D2P2F) Rabbit mAb (catalog 13647), and Histone H3 (D1H2) XP Rabbit mAb (catalog 4499). LGG was purchased from ATCC for bacterial studies. Diphtheria toxin was purchased from Fitzgerald. DSS (molecular weight 50 kDa) was purchased from Fisher. For anti–IL-10 antibody administration, 100 μg anti–IL-10 antibody (BioXcell; catalog BE0049; clone JES5-2A5) was i.p. injected into mice every other day.

### RNA-Seq and bioinformatics analysis.

The CD11b^+^ monocytes were purified from MLNs by the EasySep Mouse CD11b Positive Selection Kit (StemCell). The RNA extraction, reverse transcription, cDNA library construction and sequencing were performed by Novogene Company. The raw sequence data reported in this paper have been deposited in the Genome Sequence Archive (GSA) in National Genomics Data Center (NGDC, https://ngdc.cncb.ac.cn/) under the accession number CRA010474. Reads were mapped to mouse genome mm10 using Hisat2 software (v2.0.4). Mapped reads were assigned to mouse RefSeq genes using featureCounts and NCBI mouse annotation build 37.2. DESeq2 was used for differential gene expression analysis among treatment groups. GSEA was performed on genes ranked by their differential expression T-statistic using the clusterProfiler package in R with GO, KEGG. The hallmark gene sets were downloaded from http://software.broadinstitute.org/gsea/msigdb/genesets.jsp

### 16S rRNA gene sequencing and analysis.

Genomic DNA was extracted from colon content samples by using the CTAB/SDS method. DNA pyrosequencing of the 16S rRNA V3–V4 region was performed by Novogene Company. Briefly, the hypervariable region V3–V4 of the microbial 16S rRNA gene was amplified by PCR with indices and adaptors-linked universal primers (F: 5′-ACTCCTACGGGAGGCAGCAG-3′; R: 5′-GGACTACHVGGGTWT-CTAAT-3′). The PCR products were purified and quantified. The sequencing libraries were generated with the NEBNext Ultra IIDNA Library Prep Kit. The library was sequenced on an Illumina NovaSeq platform, and 250 bp paired-end reads were generated. The raw sequence data in the current study have been deposited in the GSA database (accession CRA010476); https://ngdc.cncb.ac.cn/gsa). The raw paired-end reads were truncated by removing the barcode and primer sequence. Paired-end reads were merged using FLASH. Quality filtering on the raw tags was performed to obtain high-quality clean tags using QIIME2 software. Diversity analysis was carried out with the counts for species level applying Vegan package in R. For each sample, bacterial Shannon and Simpson diversity were calculated using the estimate() and diversity() function of the Vegan package. β Diversity was estimated using Bray-Curtis dissimilarities between samples, which was performed by the function vegdist() in the Vegan package. Differences among samples were visualized by unconstrained PCoA, with statistical significance calculated using PERMANOVA (999 permutations) implemented by the PERMANOVA function in Primer-e. Sample scores on the PCoA axes were used as response variables in a linear mixed model. Analysis of the differential species abundance was performed using the edgeR and limma packages. *P* values were corrected for multiple tests using the approach of Benjamini and Hochberg, with α = 0.05. Linear discriminant analysis effect, implemented in LEfSe, was used to compare the relative abundance of the different taxa between LGG treatment and non-LGG treatment.

### Metabolic profiling/LC-MS-based metabolism analysis.

Approximately 100 mg of colon contents were individually grounded with liquid nitrogen, and the homogenate was resuspended with prechilled 80% methanol by well vortex. The samples were incubated on ice for 5 minutes and then were centrifuged at 15,000*g*, 4°C, for 20 minutes. Some of supernatant was diluted to final concentration containing 53% methanol by LC-MS grade water. The samples were subsequently transferred to a fresh Eppendorf tube and then were centrifuged at 15,000*g*, 4°C, for 20 minutes. Finally, the supernatant was injected into the LC-MS/MS system analysis. UHPLC-MS/MS analyses were performed using a Vanquish UHPLC system (Thermo Fisher) coupled with an Orbitrap Q ExactiveTMHF-X mass spectrometer (Thermo Fisher) using a Hypesil Gold column from Novogene Co. Ltd. The Q Exactive HF-X mass spectrometer was operated in positive/negative polarity mode to detect metabolites eluted from the column. The Compound Discoverer 3.1 (CD3.1, Thermo Fisher) software was used for the acquired MS data pretreatments. Then, the peaks were matched with the mzCloud (https://www.mzcloud.org/), mzVault, and MassList database to obtain the accurate qualitative and relative quantitative results. The KEGG and HMDB databases were used to annotate the metabolites with the exact molecular mass data (*m*/*z*) of samples. Student’s *t* test was used to detect the differences in metabolite concentrations between 2 groups. Volcano plots were used to filter metabolites of interest, which were based on log_2_(FoldChange) and –log10(*P* value) of metabolites by ggplot2 in R language. For clustering heatmaps, the data were normalized using *Z* scores of the intensity areas of differential metabolites and were plotted by Pheatmap package in R language. The correlation between differential metabolites was analyzed by cor() in R language (method = Pearson). Statistically significant of correlation between differential metabolites was calculated by cor.mtest() in R language.

### ChIP assay.

ChIP assays were conducted with a Magna ChIP A/G Chromatin Immunoprecipitation Kit (Millipore). Briefly, 5 × 10^6^ to 10 × 10^6^ bone marrow monocytes cells were fixed with a final concentration of 1% formaldehyde, cross-linked, and sonicated. The RELA antibody (10 μg/mL) (Cell Signaling Technology; catalog 8242) or IgG control antibody (Cell Signaling Technology; catalog 2729) was added to sonicated lysates, incubated overnight at 4°C, and then incubated with Protein A/G beads mixture (1:1 at ratio, CST) for another >7 hours at 4°C. Chromatin DNA was eluted, reverse cross-linked, and recovered. Input DNA and immunoprecipitated DNA were analyzed by quantitative PCR using *Il10* promoter DNA-specific primers.

### Differentiation and stimulation of bone marrow–derived monocytes.

Bone marrow was obtained from WT or DSS-treated mice and was used to prepare single-cell suspension. The cell suspension was called fresh BMCs. Marrow-derived monocytes were obtained by culture of fresh BMCs for 4 days in conditioned medium containing GM-CSF and then were further purified using the EasySep Mouse Monocyte Isolation Kit.

### ELISA.

For IL-10 ELISA assay, colon tissues were homogenized in PBS with protease inhibitor, or cell culture supernatants were obtained from monocytes. The concentration of IL-10 was measured with a Mouse IL-10 Quantikine ELISA Kit (R&D Systems) in accordance with the manufacturer’s instruction.

### Knockdown of Il10r in bone marrow monocytes.

The specific siRNA targeting mouse *il10r* was transfected into bone marrow monocytes by Lipofectamine RNAiMAX Transfection Reagent (Life Technologies) according to the manufacturer’s protocol. The sequence of siRNA (Thermo) is m*il10r* (sense): 5′-CUGGAUCUGUAUCACCGAAtt-3′. The knockdown efficiency was detected by qPCR.

### RNA extraction and RT-qPCR.

Total RNA was extracted using Trizol reagent (Life Technologies) or the RNeasy Plus Mini Kit (Qiagen) according to the manufacturer’s instructions. cDNA was prepared with the PrimeScript Reverse Transcriptase (TaKaRa). SYBR Green–based qPCR was performed using CFX96 Touch (Bio-Rad). mRNA levels were normalized to *Gadph* or *βactin* and are reported as relative mRNA expression using the Ct method.

### Statistics.

To estimate the statistical significance of differences between 2 groups, we used unpaired, 2-tailed Student’s *t* tests to calculate *P* values. A 1-way or 2-way ANOVA was performed when more than 2 groups were compared. Error bars indicate the mean ± SEM unless otherwise noted. *P* values are labeled in the figures. *P* values of less than 0.05 were considered significant. Statistical analyses were performed using GraphPad Prism (V.9.0).

### Study approval.

The mouse study was approved by the Experimental Animal Welfare and Ethical Committee of the Institute of Animal Science, Chinese Academy of Agricultural Sciences (no. IAS2021-223), and the Research Ethics Committee of the Institute of Microbiology, Chinese Academy of Sciences (no. SQ-SQIMCAS2024118).

### Data availability.

The raw sequence RNA-Seq data have been deposited in the GSA in NGDC (https://ngdc.cncb.ac.cn/; accession CRA010474). The raw sequence 16s rRNA-Seq data have been deposited in the GSA database (accession CRA010476). Values for all data points in graphs are provided in the Supporting Data Values file. Requests for any other data should be directed to and will be fulfilled by the corresponding author.

## Author contributions

LW, WS, and JZ designed the study and wrote the manuscript. WS and LW performed most of the experiments. WS performed all the bioinformatics analyses. CM, RL, YL, and Xiaohan Zhao helped to perform animal experiments and in vitro experiments. HL, Xin Zhao, and YQ provided scientific guidance for the research. HL, Xin Zhao, and JB helped to edit the manuscript.

## Supplementary Material

Supplemental data

Unedited blot and gel images

Supporting data values

## Version 1 02/03/2025

Electronic publication
