# Supplementary material for: Lactobacillus rhamnosus GG induces STING-dependent IL-10 in intestinal monocytes and alleviates inflammatory colitis in mice
Source: J Clin Invest. 2025 Feb 3;135(3):e174910. doi: 10.1172/JCI174910 (PMC11785918; doi:10.1172/JCI174910)
Supplement: Supplemental data [file jci-135-174910-s076.pdf]

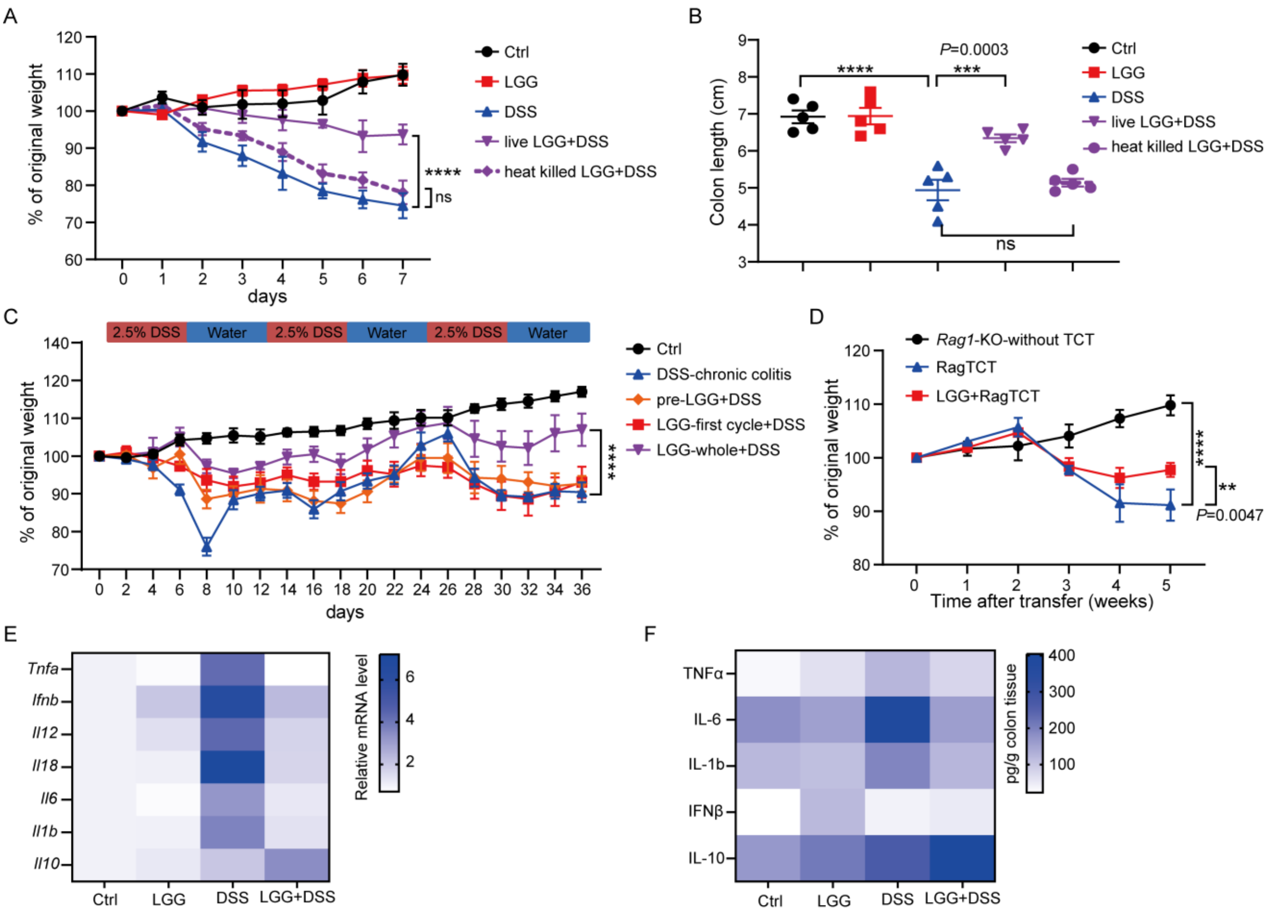

Supplemental Figure S1. Oral administration of live LGG inhibits inflammatory colitis in GF mice.

(A) Body weight assessment of wild-type GF mice receiving 3% dextran sodium sulfate (DSS) in drinking water for 7 days. WT mice were orally administered live LGG or heat killed LGG (2×10^9 CFU) daily for two weeks (starting one week before DSS administration). The body weight of mice was measured every day. The percent weight change is shown (n=5 per group).

(B) Colon length on day 7 of each group as indicated in A (n=5 per group).

(C) Experimental scheme for DSS-induced chronic colitis. DSS water (2.5%) was repetitively administered to mice to induce colitis (DSS-chronic colitis). Mice received orally administration of LGG for one week before DSS colitis induction (pre-LGG+DSS), orally administration of LGG only during the first DSS cycle (LGG-first cycle+DSS), or orally administration of LGG throughout the whole DSS-recovery cycles (LGG-whole+DSS). The body weight of mice was measured every 2 days until day 36 (n=5 per group).

(D) The $CD4^+CD45RB^{hi}CD25^-$ T cells were sorted from WT mice by flow cytometry and were transferred to Rag1 knockout mice by intraperitoneal injection ($1x10^6$ cells per mouse). Body weight assessment of *Rag1* knockout mice (*Rag1*-KO-without TCT), RagTCT mice, and RagTCT mice orally administered with LGG (2×10^9 CFU) (LGG+RagTCT). Body weight was monitored every week. The percent of body weight changes are shown (n=5 per group).

(E) Heatmap showing the mRNA expression of pro-inflammatory cytokines and anti-inflammatory cytokines genes (identified by qPCR analysis) in colon tissues of each group as indicated in A (n=3 per group).

(F) Heatmap showing the protein levels of the matching pro-inflammatory cytokines and anti-inflammatory cytokines (identified by qPCR analysis) as C (n=5 per group).

Data are expressed as mean ± SEM. One of two or three representative experiments was shown. Statistical analysis was performed using two-way ANOVA test with corrections for multiple variables (A, C, and D), and one-way ANOVA with Bonferroni's multiple comparison tests (B); ns, no significant; ***p<0.001; ****p<0.0001.

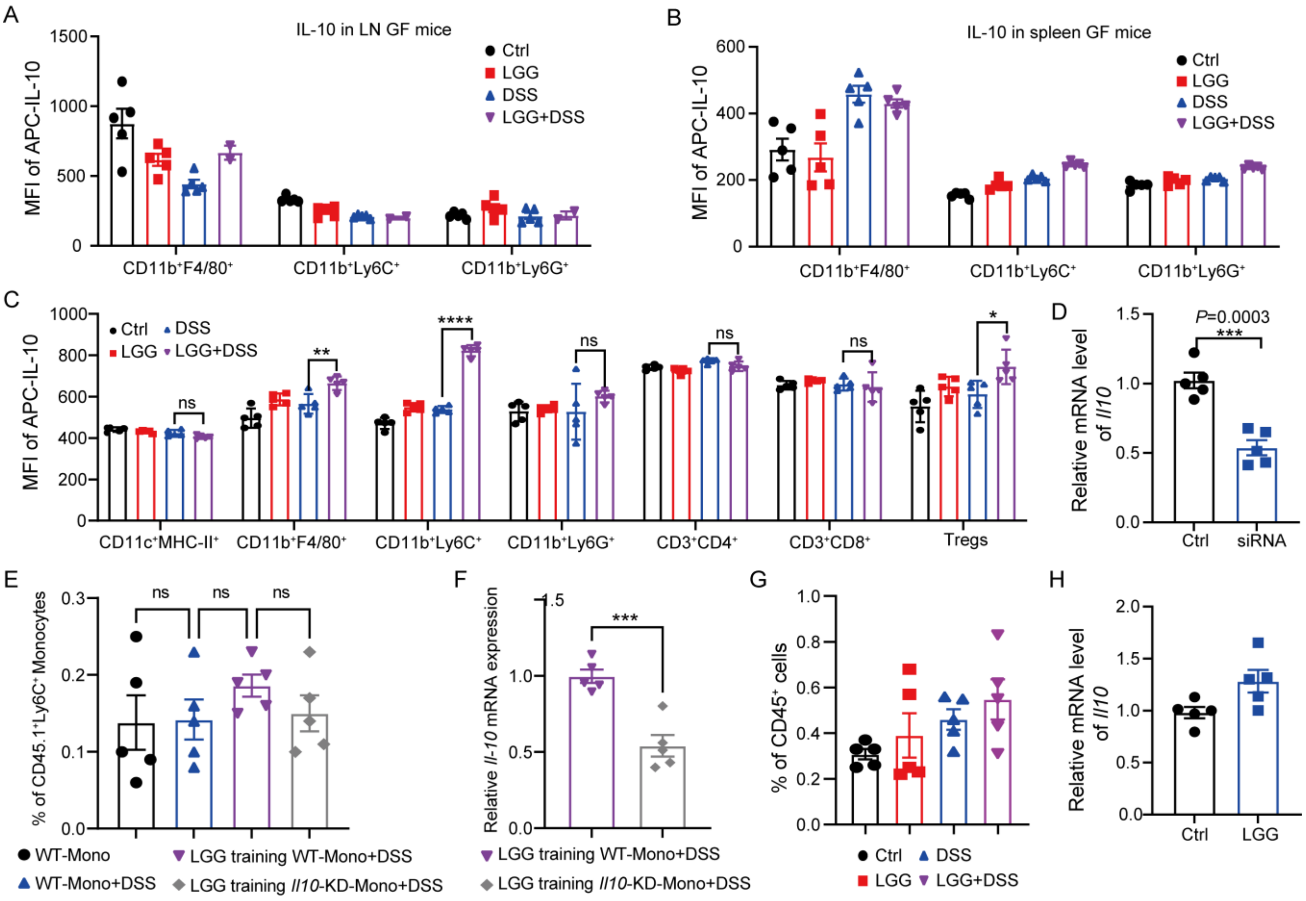

Supplemental Figure S2. Monocytic IL-10 in lymph node and spleen is not responsible for LGG-mediated protective effect against colitis.

(A-B) Mean Fluorescent Intensity (MFI) of IL-10 in different types of myeloid cells (CD45$^+$CD11b$^+$F4/80$^+$, CD45$^+$CD11b$^+$Ly6C$^+$, and CD45$^+$CD11b$^+$Ly6G$^+$) in lymph node (LN) (**A**) or spleen (**B**) from the indicated mice (n=5 per group).

(C) Mean Fluorescent Intensity (MFI) of IL-10 in different immune cells (DCs: CD11c$^+$MHC-II$^+$, Macrophages: CD11b$^+$F4/80$^+$, Ly6C$^+$ monocytes: CD11b$^+$Ly6C$^+$, Ly6G$^+$ monocytes: CD11b$^+$Ly6G$^+$, CD4$^+$ T cells, CD8$^+$ T cells, and Tregs: CD4$^+$CD25$^+$Foxp3$^+$) from lamina propria of SPF-WT mice with the indicated treatment (n= 5 mice per group).

(D) *Il10* mRNA level in BM-monocytes after the specific siRNA treatments (n=5 per group).

(E) WT or *Il10* knockdown (*Il10*-KD) bone marrow derived monocytes (CD45.1$^+$) with/without LGG treatment

were used for adoptive transfer into DSS (3%)-treated mice (CD45.2). Flow cytometry analysis of $CD45.1^+CD11b^+Ly6C^+$ monocytes in MLN from the indicated mice six days after transfer (n=5 per group).

(F) The mRNA expression of *Il10* (identified by qPCR analysis) in sorted $CD45.1^+CD11b^+Ly6C^+$ monocytes in MLN from the indicated mice six days after transfer. The qPCR data were normalized to *β-actin* (n=5 per group).

(G) Percentages of $CD11b^+Ly6C^+$ monocytes in MLN from the indicated mice (n=5 per group).

(H) qPCR analysis of *Il10* mRNA level in BM-monocytes treated with live LGG *in vitro* (n=5 per group).

Data are expressed as mean ± SEM. One of two or three representative experiments was shown. Statistical analysis was performed using one-way ANOVA with Bonferroni's multiple comparison tests (A, B, C, E, and G), and unpaired two-tailed Student's *t*-tests (D, F, and H); *p<0.05; **p<0.01; ***p<0.001; ****p<0.0001.

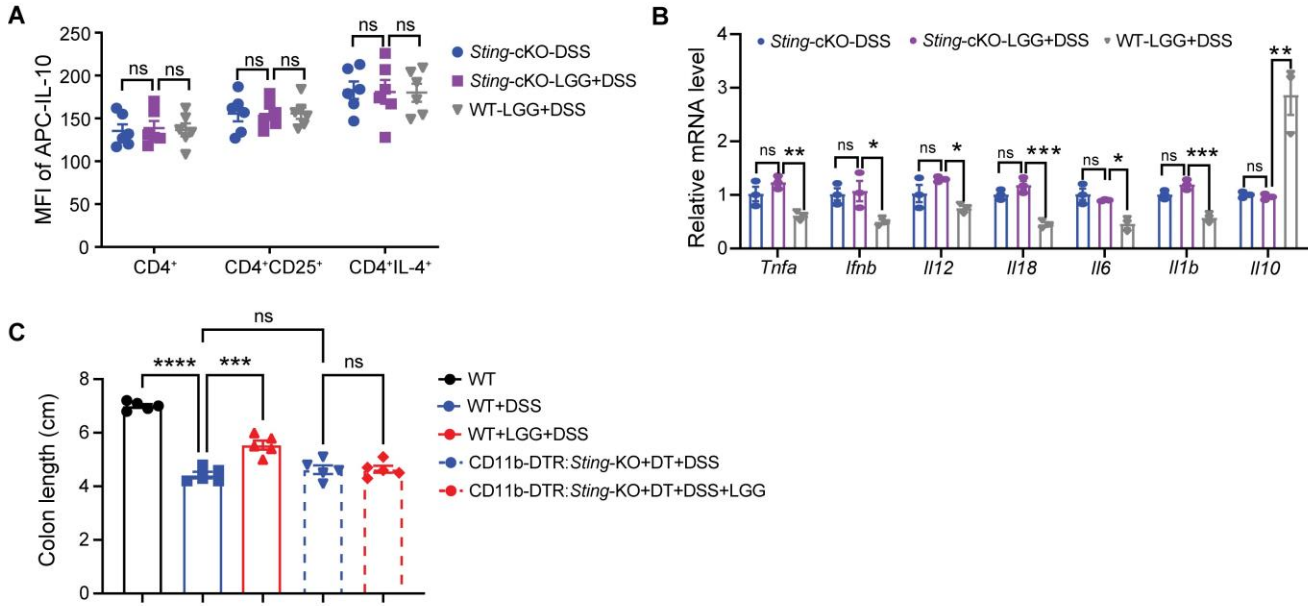

Supplemental Figure S3. LGG restrains DSS-induced colitis in a STING-dependent manner.

(A) Mean Fluorescent Intensity (MFI) of IL-10 in $CD4^+$ T cells from MLN of *Sting*-cKO or WT mice given the indicated treatment (n=6 per group).

(B) Colon tissues were collected from different mice as indicated (in A) and used for qPCR analysis of the inflammatory cytokines (n=3 per group).

(C) CD11b-DTR:*Sting*-KO bone marrow chimeric mice were injected with Diphtheria toxin (DT, 100 ng/per mouse). The next day, mice were treated with 3% DSS treatment for 7 days. For LGG treatment, mice were orally administered LGG (2×10^9 CFU) daily for two weeks (starting one week before DSS administration). The colon length of mice was measured at day 6 (n=5 per group).

Data are expressed as mean ± SEM. One of two or three representative experiments was shown. Statistical analysis was performed using one-way ANOVA with Bonferroni's multiple comparison tests (A, B, and C); ****p*<0.001.

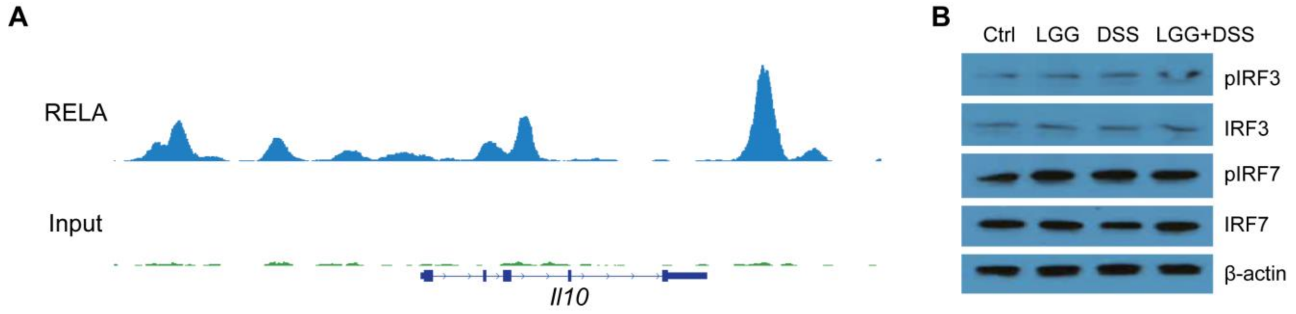

Supplemental Figure S4. LGG activates a STING/TBK1/RELA/IL-10 axis in monocytes during colitis.

(A) Profile of RELA binding (GSE99895) at the promoter region of *Il10* in bone marrow-derived macrophages.

(B) Ly6C$^+$ monocytes were collected from the MLN in WT mice with DSS with/without LGG treatment. Graph showing the immunoblot analysis of different proteins as indicated in either total levels or phosphorylation levels. One of two representative experiments was shown (B).

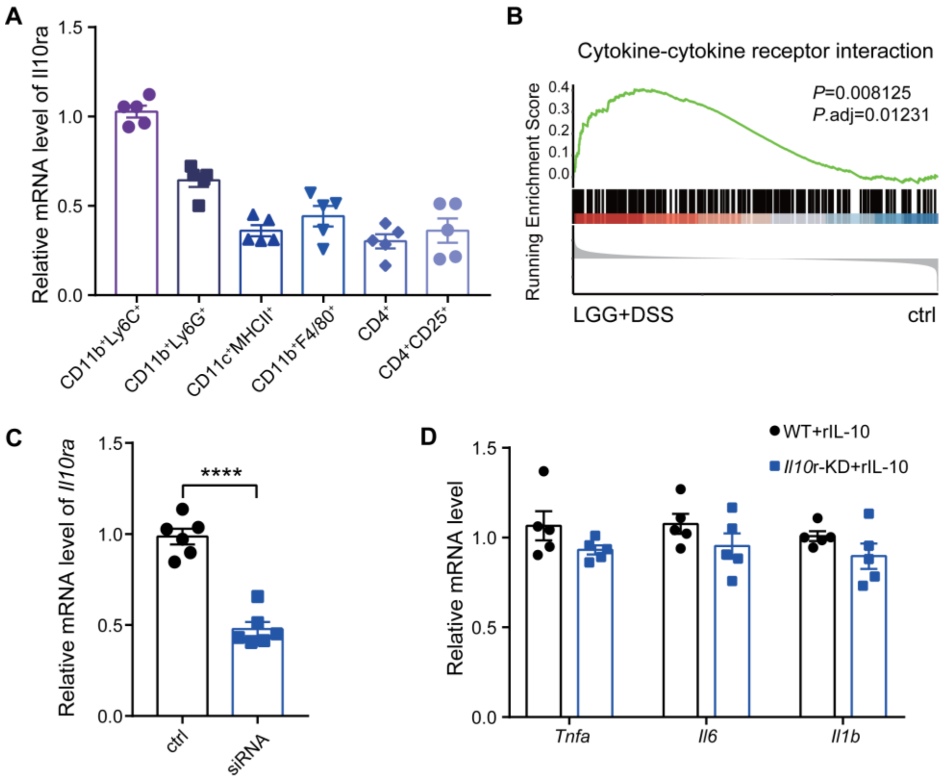

Supplemental Figure S5. LGG triggers an IL-10-based autocrine regulatory loop in monocytes during colitis.

(A) Immune cells sorted from the MLN of LGG+DSS treated WT mice at day 7 and used for qPCR analysis of *Il10ra* (n=5 per group).

(B) Gene set enrichment analysis plots of "cytokine-cytokine receptor interaction" positively correlated with LGG+DSS treatment based on the RNA-seq data of CD11b$^+$ monocytes isolated from MLN.

(C) *Il10ra* mRNA level in BM-monocytes after the indicated siRNA treatment (n=6 per group).

(D) Ly6C$^+$ monocytes isolated from LGG+DSS treated mice and stimulated with recombinant IL-10 (10 ng/mL) and qPCR analyses of the mRNA levels of *Tnfa*, *Il6*, and *Il1b* (n=5 per group).

Data are expressed as mean ± SEM. One of two representative experiments was shown. Statistical analysis was performed using unpaired two-tailed Student's *t*-tests (C, and D); ****p<0.0001.

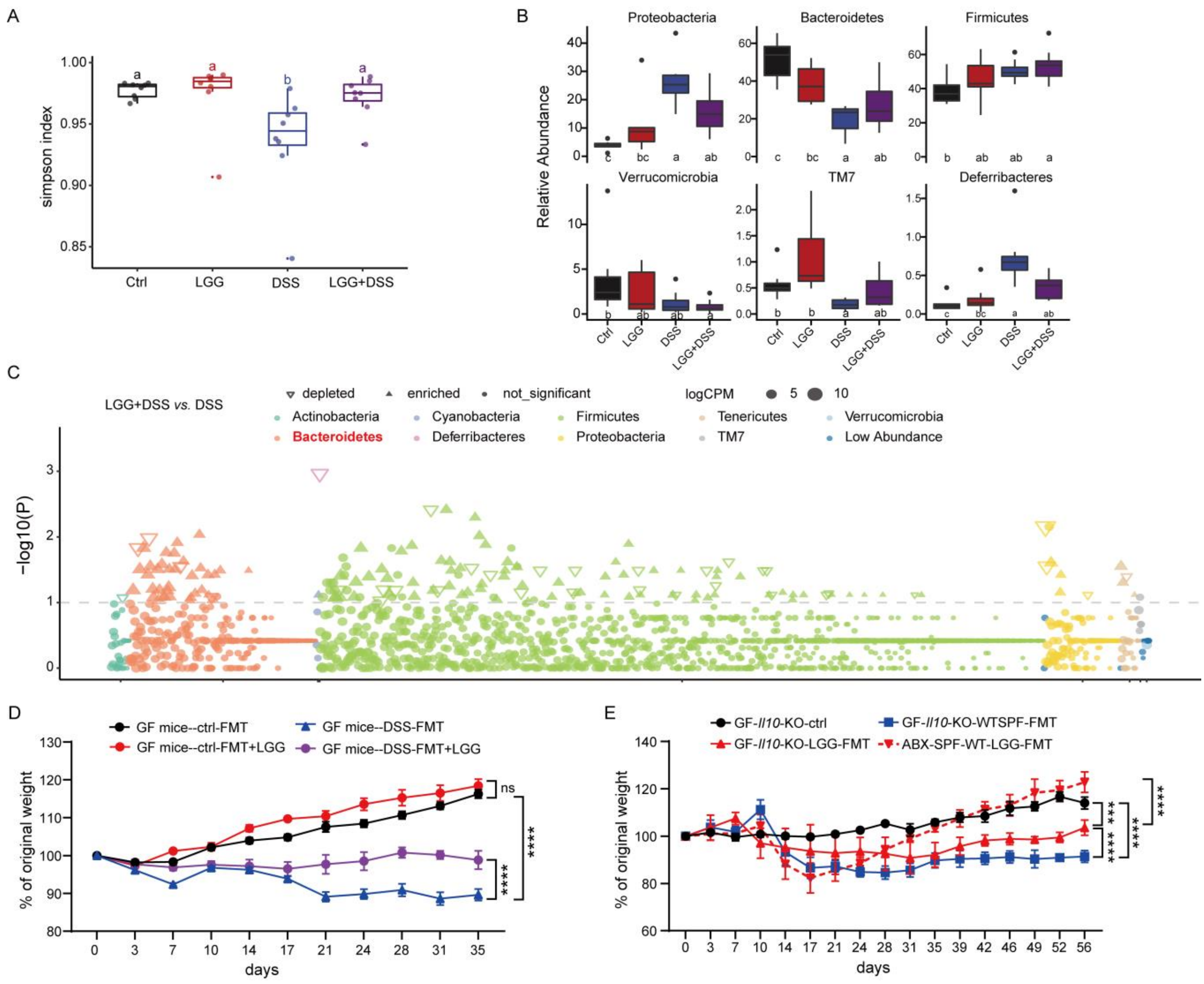

Supplemental Figure S6. LGG shapes the gut microbial community associated with intestinal immune responses.

(A) Boxplots of Simpson diversity index for each treatment groups. DSS treatment group shows lower diversity. Alpha diversity scores calculated by subsampling samples to 11000 reads with 1000 iterations. Each point represents the diversity score for one sample. Between-group variations were measured using ANOVA and T-test. All p values were adjusted using the Benjamini and Hochberg methods.

(B) Comparison of relative abundance of bacterial phylum in the different groups. Median abundances (horizontal line) and interquartile ranges have been indicated in the plots. Relative abundance of *Proteobacteria*, *Bacteroidetes*, *TM7*, *Deferribacteres* (phylum level) exhibit significant (Kruskal Wallis test, BH corrected

$p < 0.05$) differential abundance in the gut microbiomes from ctrl, LGG, DSS and LGG+DSS groups.

(C) Manhattan plot showing ASVs enriched in either the combination treatment group or DSS treatment group. Each dot or triangle represents a single ASV. ASVs enriched in the combination treatment group are represented by filled triangles; for DSS treatment group: empty triangles (FDR adjusted $p<0.05$ and log2fold change >1, Wilcoxon rank sum test). ASVs are arranged at the phylum level and colored according to the phylum. CPM, counts per million.

(D) FMT was performed with fresh feces from WT SPF mice without any treatment (ctrl) or DSS-treated WT SPF mice (with 3%-DSS treatment for six days) via oral gavage twice weekly over five weeks. Mice were orally administered with LGG (2×10^9 CFU) twice weekly for five weeks (starting at the same day with FMT). Body weight was monitored twice one week (n=5 per group).

(E) FMT was performed with fresh feces from non-LGG (GF-*Il10*-KO-WTSPF-FMT) or LGG (GF-*Il10*-KO-LGG-FMT) training mice into germ-free *Il10*-KO mice weekly over 7 weeks. FMT was performed with fresh feces from LGG training mice (ABX-SPF-WT-LGG-FMT) into ABX-treated WT mice as positive control. Body weight was monitored twice one week (n=5 per group).

Data are expressed as mean ± SEM. Statistical analysis was performed using two-way ANOVA test with corrections for multiple variables (D, and E). *$P < 0.05$, **$P < 0.01$, ***$P < 0.001$, and ****$P < 0.0001$.

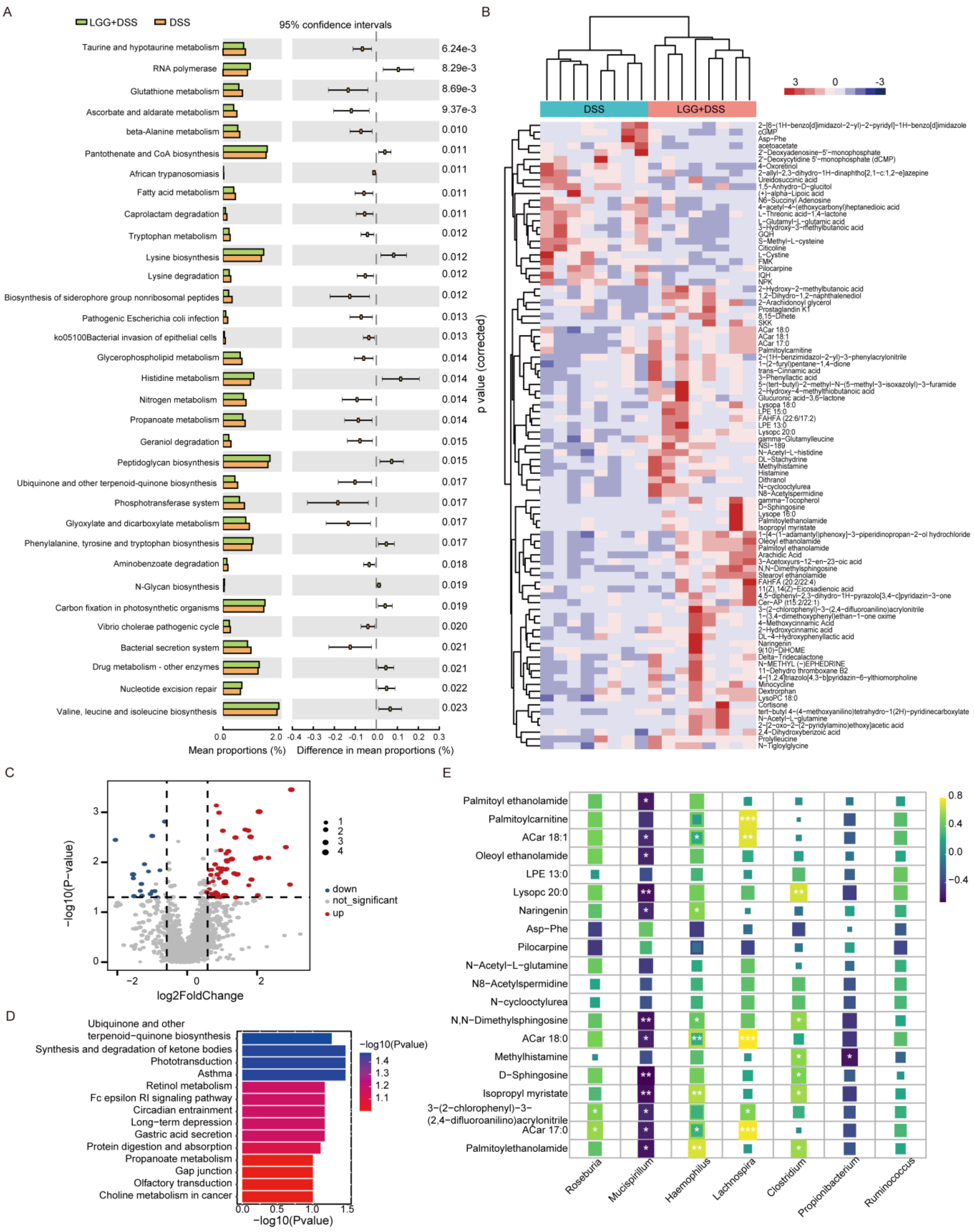

Supplemental Figure S7. The metabolic function of LGG-reshaped gut microbiome.

(A) Microbial functional prediction by PICRUSt of the gut microbiome in DSS and LGG+DSS treatment groups

with reference to the KEGG database level 2. Statistical analysis was carried out using STAMP software.

(B) Heatmap provides intuitive visualization of 89 top-ranking metabolites from the partial least squares discriminant analysis variable importance in projection (PLS-DA VIP). Each column represents a sample and each row represents a metabolite. Colors on the column caps depict sample type (blue= DSS, red = LGG+DSS). Metabolites are represented on the right-hand side of the plot. The color (from blue to red) represents the expression profile of metabolite from low to high.

(C) Volcano plot showing the significantly different metabolites between DSS treatment group and LGG+DSS treatment group. Each point represents a metabolite, Red, blue, and gray represent the upregulated, downregulated, and non-significant changes, respectively. Larger symbol sizes represent higher VIP values.

(D) KEGG enrichment analysis of differential metabolites in the intestine. Bar graphs of the top fourteen most enriched metabolic pathways.

(E) Correlation analysis of the microbiome and metabolome in DSS and LGG+DSS treatment groups. PLS-DA statistics were used for the relative abundance of differential metabolites. Differentially abundant taxa as identified by LEfSe analysis. The color was according to the Spearman correlation coefficient distribution. Yellow represented significant positive correlation; blue represented significantly negative correlation. White stars within boxes indicate significant results (*$p < 0.05$; **$p < 0.01$, Student's t test (two-sided), Benjamini–Hochberg adjustment for multiple comparisons).
