# Supplementary material for: Lactobacillus rhamnosus GG induces STING-dependent IL-10 in intestinal monocytes and alleviates inflammatory colitis in mice
Source: J Clin Invest. 2025 Feb 3;135(3):e174910. doi: 10.1172/JCI174910 (PMC11785918; doi:10.1172/JCI174910)
Supplement: Unedited blot and gel images [file jci-135-174910-s077.pdf]

Full unedited blot/gel for Figure. 4

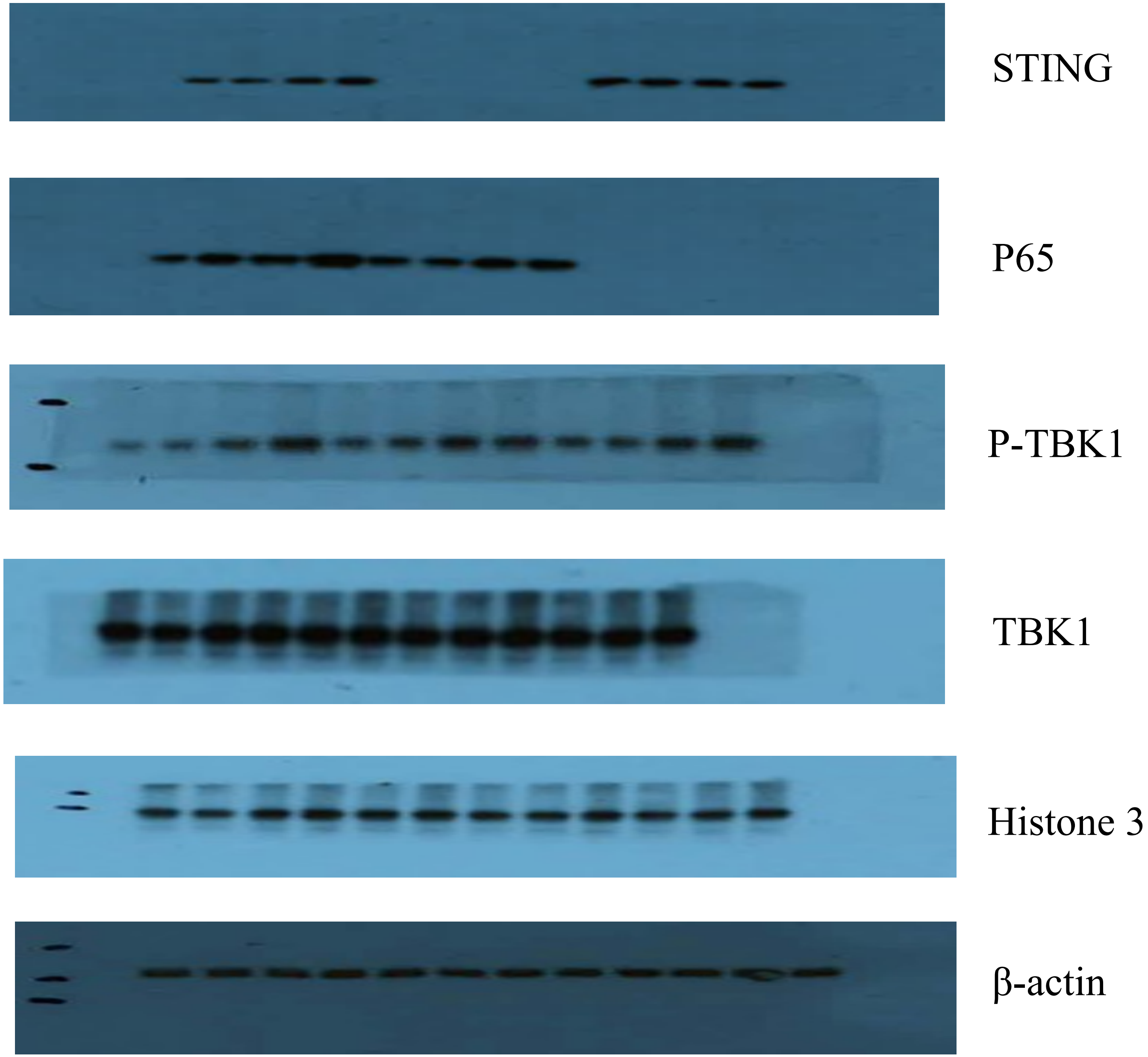

Full unedited blot/gel for Figure S4

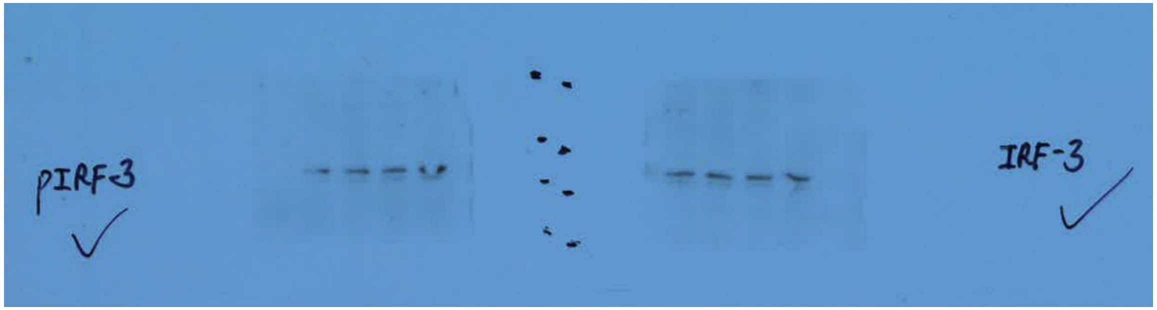

IRF3 and p-IRF3

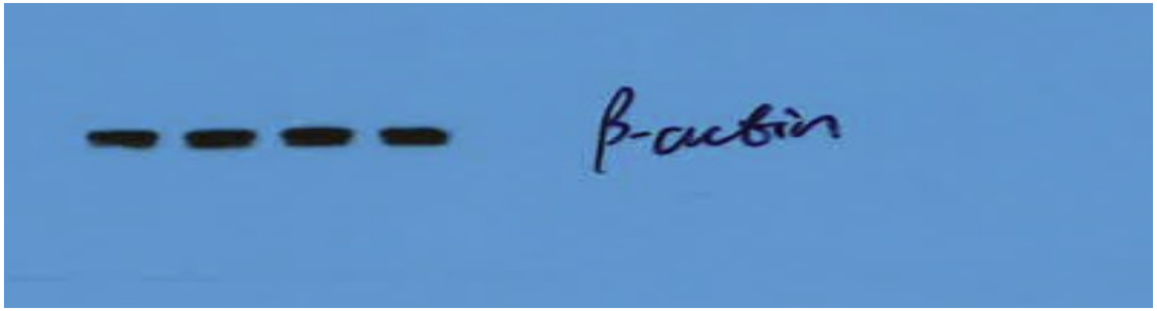

β-actin

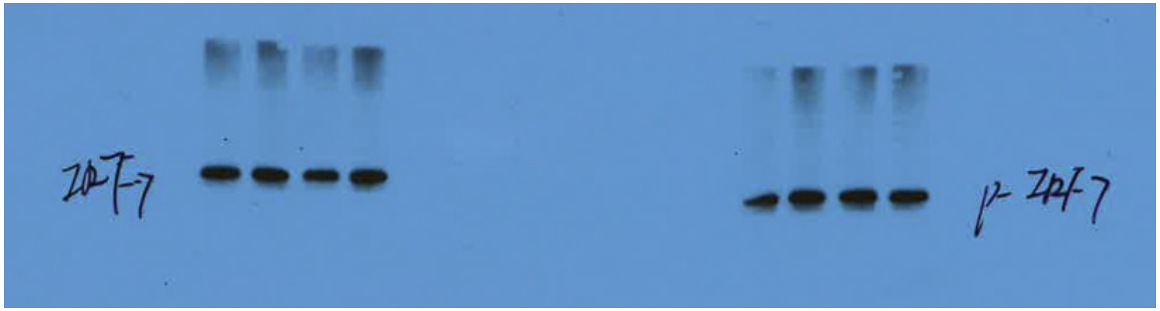

IRF7 and p-IRF7
